# Homodimerized cytoplasmic domain of PD-L1 regulates its complex glycosylation in living cells

**DOI:** 10.1038/s42003-022-03845-4

**Published:** 2022-08-30

**Authors:** Li Zhou, Fangni Chai, Yong He, Zhihui Zhou, Shupan Guo, Pan Li, Qi Sun, Xueyin Zu, Xin Liu, Qin Huang, Yanping Zhong, Aolan Zhou, Xueyun Wang, Haiyan Ren

**Affiliations:** 1 Division of Respiratory and Critical Care Medicine, State Key Laboratory of Biotherapy, West China Hospital of Sichuan University, 610041 Chengdu, China; 2 Collaborative Innovation Center of Biotherapy, 610041 Chengdu, China

**Keywords:** Membrane proteins, Glycosylation, Synthetic biology

## Abstract

Whether membrane-anchored PD-L1 homodimerizes in living cells is controversial. The biological significance of the homodimer waits to be expeditiously explored. However, characterization of the membrane-anchored full-length PD-L1 homodimer is challenging, and unconventional approaches are needed. By using genetically incorporated crosslinkers, we showed that full length PD-L1 forms homodimers and tetramers in living cells. Importantly, the homodimerized intracellular domains of PD-L1 play critical roles in its complex glycosylation. Further analysis identified three key arginine residues in the intracellular domain of PD-L1 as the regulating unit. In the PD-L1/PD-L1-3RE homodimer, mutations result in a decrease in the membrane abundance and an increase in the Golgi of wild-type PD-L1. Notably, PD-1 binding to abnormally glycosylated PD-L1 on cancer cells was attenuated, and subsequent T-cell induced toxicity increased. Collectively, our study demonstrated that PD-L1 indeed forms homodimers in cells, and the homodimers play important roles in PD-L1 complex glycosylation and T-cell mediated toxicity.

## Abstract (web summary)

Membrane-anchored PD-L1 homodimerizes in cells. N-glycosylation of PD-L1 regulates its homodimerization. The formation of PD-L1 homodimers further regulates their glycan processing, localization to the plasma membrane, and binding to PD-1.

## Introduction

Programmed death-1 ligand 1 (PD-L1) is frequently highly expressed on the surface of different tumor cells, such as lymphoma, melanoma, lung and breast cancer^1, 2^. PD-L1 binding with its receptor PD-1 inhibits T-cell function and facilitates tumor cell escape from the immune system^3, 4^. Antibodies targeting the PD-1/PD-L1 immune checkpoint have been used with remarkable success in clinical practice^5–12^. However, the response rate of patients receiving PD-1/PD-L1-inhibiting monotherapy rarely exceeds 40%. In addition, a considerable number of patients present with resistance to PD-1/PD-L1 therapies^13^. Moreover, the detection of PD-L1 in patient samples does not lead to consistently accurate predictions of anti-PD-1/PD-L1 therapeutic efficacy. Hence, new strategies and combination therapies, as well as improved predictive assays, are needed. An in-depth understanding of the regulatory mechanism of the PD-1/PD-L1 axis may potentially lead to novel strategies and facilitate accurate clinical prognostics of related cancer therapy.

As a member of the B7 family, PD-L1 consists of an immunoglobulin V-like domain (IgV, F19-T127), an immunoglobulin C-like domain (IgC, P133-V225), a transmembrane domain (TM, T239-F259) and a short intracellular tail (R260-T290)^14, 15^. Bristol-Myers Squibb (BMS) compound-mediated PD-L1 homodimerization of the extracellular domain has been observed in size exclusion chromatography, nuclear magnetic resonance (NMR) analysis, and crystal structure^16^. The free extracellular domain (ECD) of PD-L1 has been suggested to form weak homodimers through eight hydrogen bonds, as ascertained by studying the crystal structure^17^. However, it remains unclear whether the PD-L1 homodimer forms under physiological conditions and whether such a homodimer plays a role in immunological synapses. Full-length PD-L1 anchored to the cell surface may behave differently from the ECD in vitro. Furthermore, PD-L1 is subject to abundant posttranslational modifications that play important physiological roles^18–22^. PD-L1 modification, such as glycosylation, may create spatial conformational hindrances and inhibit PD-L1 homodimerization in cells. In addition, the physiological significance of PD-L1 homodimers is unknown, which attracted our intense attention.

Here, we genetically introduced cross-linker into specific positions of the PD-L1 protein, which could irreversibly lock protein interactions with high specificity, reliability, and accuracy. With the help of photocross-linking unnatural amino acids (UAAs) and proximity-enabled bioreactive UAAs^23–28^, we demonstrated that full-length human PD-L1 (hPD-L1) forms asymmetric homodimers and tetramers through the transmembrane domain (TM), extracellular domain (ECD) and intracellular domain (ITD) in living cells. Our in-cell study revealed that initiation of N-linked glycosylation is important for PD-L1 homodimerization. Importantly, dimerization of PD-L1 ITD plays a critical role in the complex glycosylation of PD-L1. In addition, we mapped homodimerized key arginine residues (R260/R262/R265) in the intracellular domain as functional units to regulate PD-L1 complex glycosylation. In the PDL-1/PD-L1-3RE homodimer, mutations of the arginine result in its plasma membrane localization decreasing and Golgi accumulation increasing of wild-type (WT) PD-L1. Notably, regulating PD-L1 glycosylation shows important clinical implications^13, 29, 30^. Highly mannosylated PD-L1 in defective PD-L1 homodimer shows attenuated PD-1 binding ability and enhanced T-cell-induced toxicity. Altogether, we verified that PD-L1 homodimerizes in living cells and revealed its critical role in PD-L1 complex glycosylation and related function.

## Results

### PD-L1 homodimerized in living cells

**Fig. 1:**
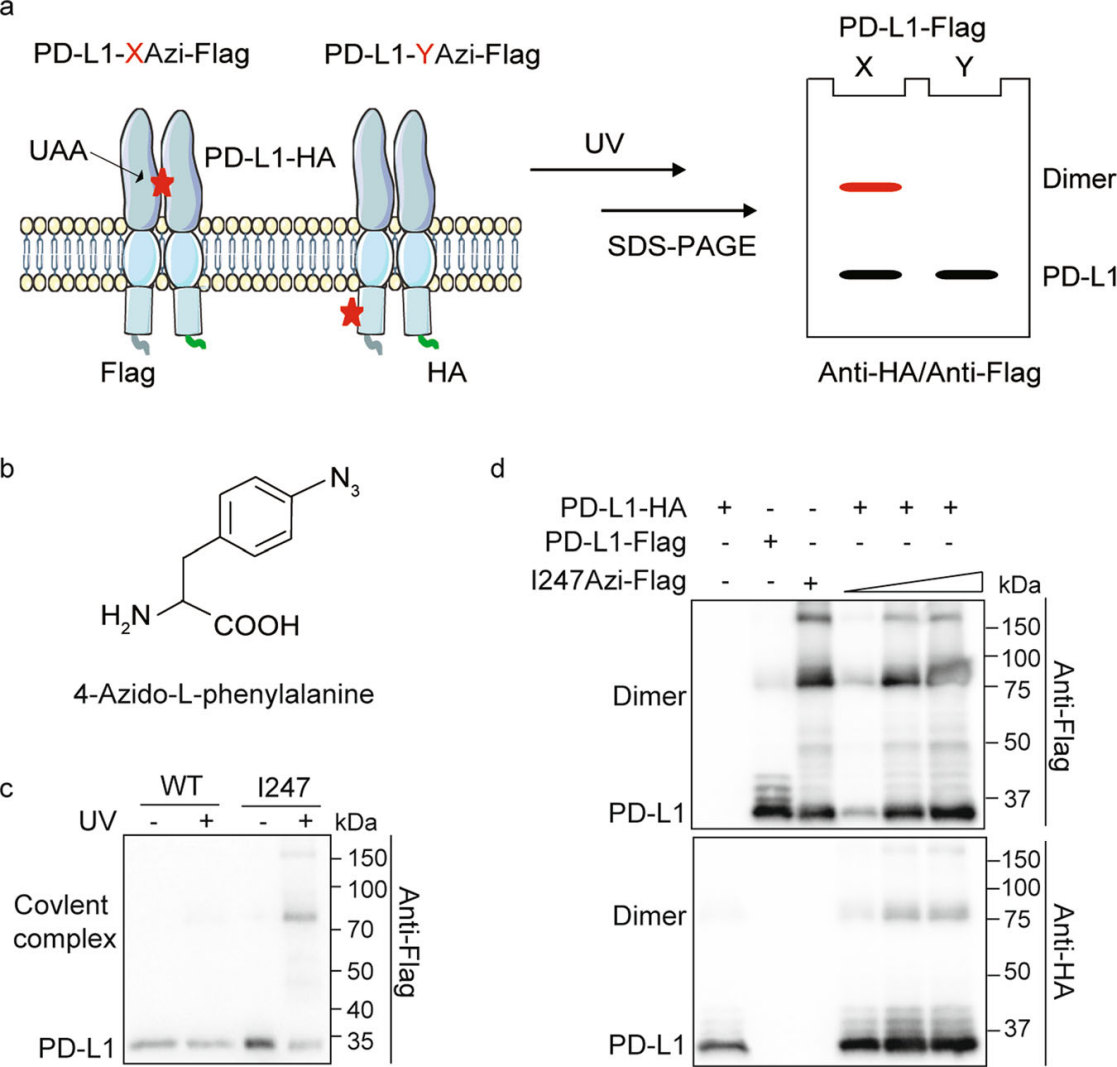
PD-L1 homodimerized in living cells. **a** Schematic diagram of genetically incorporated photocross-linking UAAs covalent capture PD-L1 homodimer. UAAs incorporated positions were referred with *X* or *Y*. PD-L1-X/YAzi-Flag, and WT PD-L1-HA were coexpressed in living cells. The anti-HA positive cross-linking band (colored red) is the complex of PD-L1-HA captured by PD-L1-XAzi-Flag. Red stars indicated the incorporated UAAs. Green curves indicated the HA tag and light blue curves indicated the Flag tag. **b** Structural formula of the 4-Azido-l-phenylalanine (Azi). **c**, **d** PD-L1-I247Azi captures homodimer dependent on the UAA incorporation and UV treatment. **c** Cells expressing PD-L1-I247Azi capture cross-linking bands in correspondence to PD-L1 homodimer. HEK293T cells were transiently transfected with PD-L1-I247tag-Flag and pIRE4-Azi plasmids. 1 mM of Azi was added to the medium. Cell lysates were separated on SDS-PAGE gels. Immunoblotting was performed using anti-Flag antibodies. **d** HEK293T cells were cotransfected with PD-L1-I247tag-Flag, pIRE4-Azi and PD-L1-HA plasmids. The quantity of PD-L1-I247tag-Flag plasmid increased from lane 4 to lane 6. All samples were treated with PNGase F to remove the N-glycan.

Homodimerization of proteins plays a key role in many cellular processes^31–41^. PD-L1 homodimerization and the functions of PD-L1 homodimers have not been explored in living cells. Our strategy to capture PD-L1 homodimers in mammalian cells is based on the genetic incorporation of reactive unnatural amino acids into specific positions of the hPD-L1 protein (Fig. 1a). The photocross-linking UAAs form cross-linking moiety upon ultraviolet (UV) irradiation and covalently capture natural amino acids of proximal interaction partners (Fig. 1a). The covalent complexes are detected by the molecular weight (MW) of the denatured adduct in a denatured PAGE gel (Fig. 1a).

Using the synthetase-tRNA_AUC_ suppression system, the photocross-linking UAA, 4-Azido-L-phenylalanine (Azi, Fig. 1b), was genetically incorporated into a specific position of the PD-L1-Flag protein corresponding to the amber stop codon (TAG) in HEK293T cells (Supplementary Fig. 1a). We achieved yields of PD-L1-Azi mutants up to 88-100% of WT PD-L1 (Supplementary Fig. 1b). Intact cells expressing PD-L1-I247Azi were irradiated with UV light to promote cross-linking, and a band (~75 kDa) corresponding to the PD-L1 homodimer was detected (Fig. 1c). The formation of the adduct band is UAA incorporation and UV-dependent (Fig. 1c). To test whether the band formed after cross-linking is a PD-L1 homodimer, we coexpressed PD-L1-I247Azi with a Flag tag and WT PD-L1 with a hemagglutinin (HA) tag in cells. Immunoblot analysis with an anti-HA antibody showed a signal at 75 kDa at all the PD-L1-I247Azi expression levels analyzed, indicating full-length PD-L1 homodimerized in living cells (Fig. 1d). The covalent complexes were also identified with an anti-PD-L1 antibody (Supplementary Fig. 1c). To illustrate that the bands generated after cross-linking are specific, we also tested the complexes formation of other PD-L1 mutants with Azi residues (PD-L1-XAzi). Only certain PD-L1-XAzi samples showed cross-linking bands corresponding to homodimers under UV irradiation (Supplementary Fig. 1d). Overall, our results indicated that full-length PD-L1 homodimerized in living cells.

### PD-L1 homodimerized/tetramerized through the transmembrane domain and extracellular domain

**Fig. 2:**
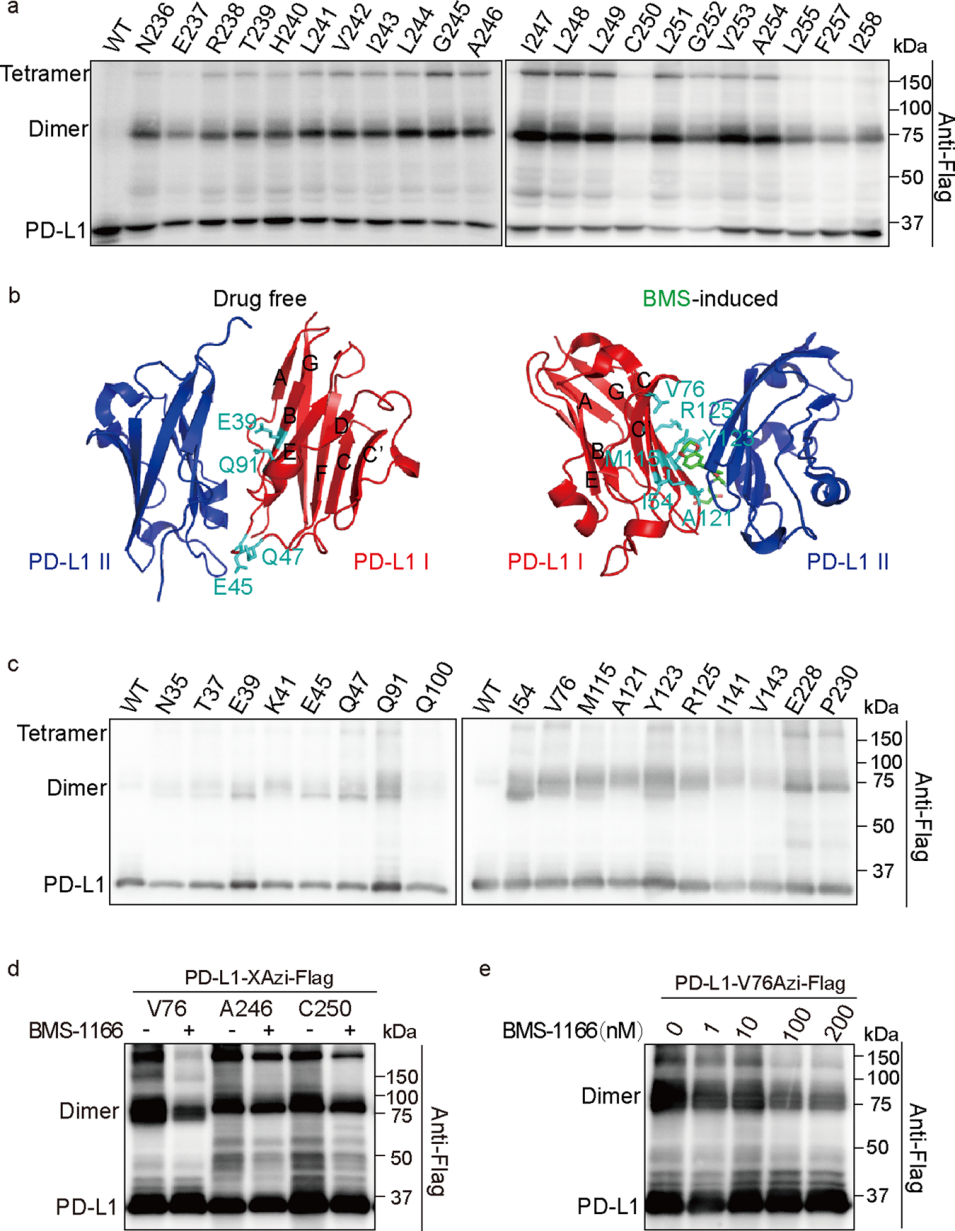
The transmembrane domain and extracellular domain of PD-L1 are involved in homodimerization. **a** Transmembrane domain of PD-L1 is involved in its homodimerization. Residues that were replaced by Azi are indicated in the upper row. WT PD-L1 treated with UV was used as a control. HEK293T cells were irradiated with UV before being lysed. Shown are immunoblotting using anti-Flag antibodies. **b** Structure of drug-free and BMS-induced PD-L1 homodimer. The PDB ID is 3FN3 for drug-free PD-L1 homodimer and 5N2F for BMS-induced PD-L1 homodimer. BMS was colored green. Residues that are important for PD-L1 homodimerization were colored cyan. **c** Screening of residues in extracellular domain that covalent captured PD-L1 homodimer. Transfected cells expressing PD-L1-XAzi-Flag proteins were treated with UV to induce cross-linking and performed immunoblotting analysis. **d**, **e** BMS-1166 reduces Azi-mediated covalent capture of PD-L1 homodimerization dosage dependently. HEK293T cells were transiently transfected with PD-L1-Xtag-Flag and pIRE4-Azi plasmids. **d** Residues replaced by Azi are indicated in the upper row. 100 nM of BMS-1166 were incubated with cells for 18 h before UV treatment. **e** Working concentration of BMS-1166 ranged from 0 to 200 nM. All samples were treated with PNGase F to remove the N-glycan.

UAAs were genetically incorporated into specific residues corresponding to TAG stop codons (Supplementary Fig. 1a). The TAG context results in the variation of UAA incorporation rates in bacteria and mammalian cells^42–44^. The incorporated UAAs cross-link interacting proteins only when they are located at the protein-protein interaction surface. The fidelity of UAA incorporation and the specificity of UV cross-linking emphasize the value of this method for addressing protein-protein interaction surfaces. To understand the molecular basis of PD-L1 homodimerization, we incorporated UAAs into different positions of PD-L1 and found that most residues in the PD-L1 TM (T239-F259) are involved in PD-L1 homodimerization (Fig. 2a). However, the cross-linking mediated by some residues, such as C250, L255, and F257, was weak (Fig. 2a). Residues on more than one TM surface involved in this interaction may be caused by PD-L1 polymerization or asymmetrical homodimerization. A previous NMR analysis indicated that N-terminal human PD-L1(A18-Y134) loosely forms tetramer in solution induced by BMS small molecules^45^. However, size exclusion chromatography and X-ray analysis indicated that the most stable BMS-induced oligomerized PD-L1 unit is a dimer^45^. These results may be explained by the native transient tetrameric state or the use of truncated PD-L1 in the experiments. In addition, free hPD-L1 in crystal models has been shown to comprise two contacting molecules, an asymmetric dimer^17^. Since a cross-linking band corresponding to a PD-L1 tetramer was observed in our site-specific covalent cross-linking experiments (Fig. 2a), we suggest that full-length hPD-L1 proteins in living cells form homodimer/tetramers through the TM domain.

The ECDs involved in natural^17^ and BMS-induced PD-L1 homodimerization^45^ differ (Fig. 2b). Therefore, we selectively replaced relevant residues in the extracellular IgV domain of PD-L1 (F19-T127) with Azi. Residues at the interaction interface of the native homodimer (E39, E45, Q47, and Q91) and on the interaction interface of the BMS-induced homodimer (I54, V76, M115, A121, Y123, and R125) were involved in PD-L1 homodimerization under physiological conditions (without BMS) (Fig. 2b, c). Overall, Azi-replaced residues on the interaction interface of the BMS-induced homodimer show stronger cross-linking (Supplementary Fig. 2a). We also tested PD-L1 dimerization in the presence of BMS-1166, a high-performance BMS compound. Surprisingly, BMS-1166 attenuated the covalent bond formation of the PD-L1 homodimer in a dose-dependent manner (Fig. 2d, e), which may be caused by conformational changes induced by BMS-1166. Moreover, E228 and P230 in the flexible linker (FL) between the PD-L1 TM domain and ECD are also involved in PD-L1 homodimerization (Fig. 2c). However, no obvious covalent homodimers were found when the UAAs were incorporated into any residues of the intracellular domain (F259-T290) (Supplementary Fig. 2b, c). In conclusion, PD-L1 homodimerizes and tetramerizes through the TM domain and ETD in living cells. However, we cannot rule out the possibility that the ITD of PD-L1 homodimerized.

### Pairwise chemical cross-linking reveals that PD-L1 is asymmetrically homodimerized through the TM domain, ITD, and ECD

**Fig. 3:**
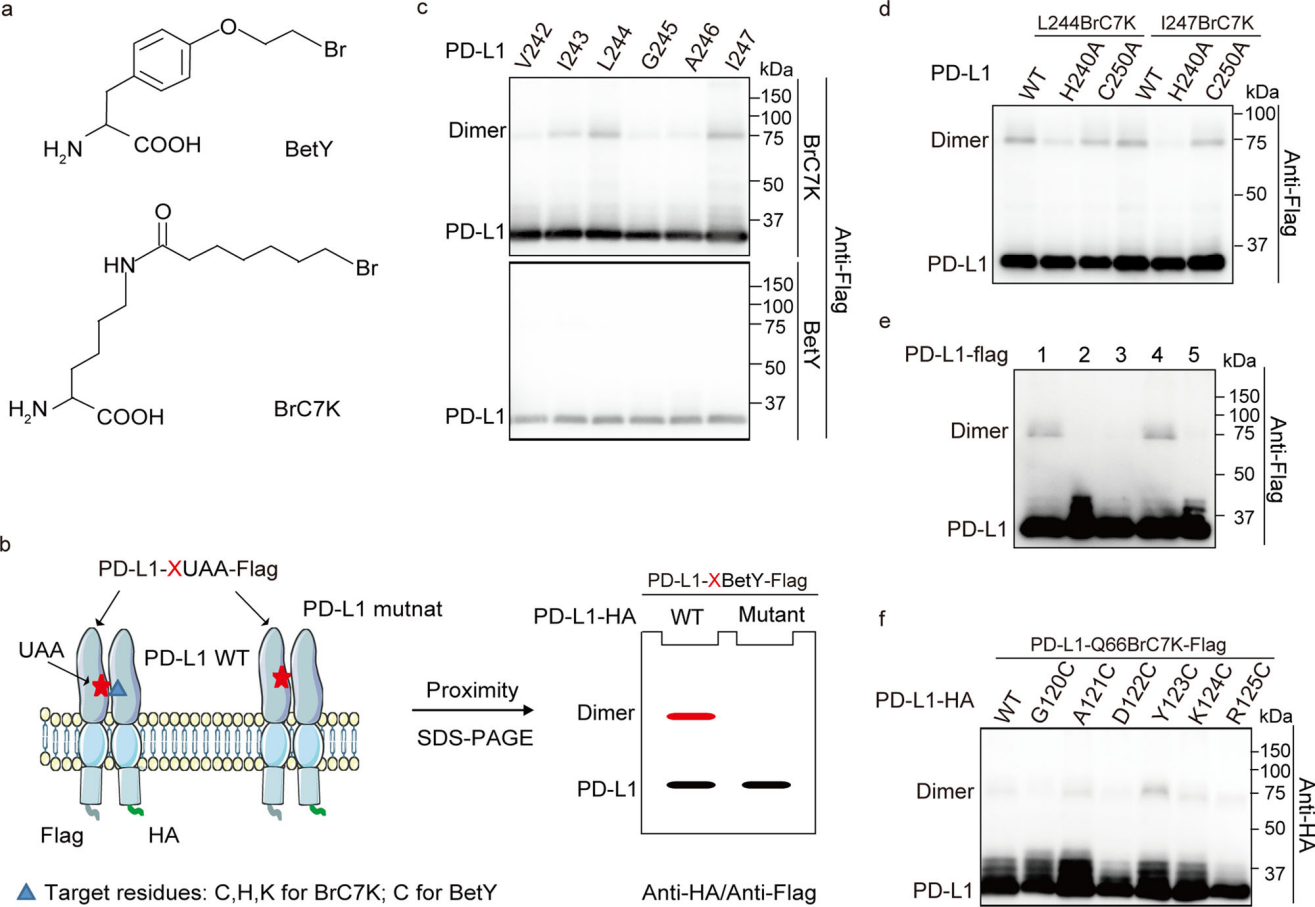
PD-L1 asymmetrically homodimerized. **a** Structural formula of proximity-enabled UAAs, BrC7K, and BetY. **b** Schematic diagram of mapping interacting residue pairs using the chemical cross-linking UAAs. Red stars represent the proximity-enabled UAAs, namely BrC7K/BetY. BrC7K/BetY selectively reacts with targeted amino acids (blue triangle) to form covalent complexes which are destroyed by targeted amino acids mutation. **c** BrC7K incorporated into the transmembrane domain of PD-L1 capture its homodimers. PD-L1-Xtag-Flag plasmid was cotransfected with pHY-XYRS or pHY-BrC7KRS to incorporate the BetY or BrC7K individually. Samples were collected for anti-Flag immunoblotting analysis. Residues replaced by BrC7K/BetY are indicated in the upper row. **d** BrC7K at L244 and I247 of PD-L1 interact with H240 residues. PD-L1-L244/I247BrC7K-Flag with or without the H240A/C250A mutation were expressed in HEK293T cells. **e** D284 in the intracellular domain of PD-L1 interacts with C272. BrC7K were incorporated into D284 of PD-L1 or PD-L1 mutants. Lane 1: PD-L1-D284BrC7K-Flag; Lane 2: PD-L1-C272A-Flag; Lane 3: PD-L1-C272A-D284BrC7K-Flag; Lane 4: PD-L1-D284BrC7K-H286A-Flag; Lane 5: PD-L1-Flag. **f** Q66 and Y123 in the extracellular domain of PD-L1 are paired interaction residues. Residues mutated to Cys are indicated in the upper row. All samples were treated with PNGase F to remove the N-glycan.

We then focused on deciphering further details of the PD-L1 homodimer using pairwise chemical cross-linking. The proximity-enabled UAAs BrC7K and BetY selectively react with targeted residues (Cys, His, and Lys for BrC7K, Cys for BetY) when they are in close proximity and in the correct orientation (Fig. 3a)^24, 46^. Mutation of the targeted amino acid prevents the formation of the covalent bond, which allows the interacting amino acid pairs to be identified (Fig. 3b)^47^. Therefore, we sought to identify interacting residue pairs in PD-L1 homodimers using proximity-enabled cross-linking UAAs. First, BrC7K (Supplementary Fig. 3a) and BetY were genetically incorporated into the transmembrane domain of PD-L1. The cross-linked PD-L1 homodimer was identified in PD-L1-I243/L244/I247BrC7K samples but not in samples carrying PD-L1-BetY (Fig. 3c). These results, together with sequence analyses, suggest that H240 and C250 in PD-L1 TM are the potential targets (Supplementary Fig. 3b). As expected, the H240A mutation, but not the C250A mutation, destroyed the formation of the covalent PD-L1-L244BrC7K homodimer and PD-L1-I247BrC7K homodimer (Fig. 3d). Photocross-linking experiments confirmed that PD-L1-H240A and PD-L1-C250A mutants retained homodimerization activity (Supplementary Fig. 3c, d). We also replaced individual residues near H240 with cysteine residues. Among these mutants, the PD-L1-T239C and PD-L1-H240C mutants showed the most intense cross-linking signals (Supplementary Fig. 3e). Moreover, BrC7K incorporation into the ITD of PD-L1 revealed that PD-L1-D284BrC7K also captures the PD-L1 homodimer (Supplementary Fig. 3f). Target residue mutation scanning revealed that D284 reacts with C272 in living cells (Fig. 3e and Supplementary Fig. 3f). Therefore, PD-L1 asymmetrically homodimerizes through the TM domain and the ITD.

We then focused on pinpointing the residue pairs involved in intermolecular interactions of the PD-L1 ECD using pairwise chemical cross-linking. The cysteine residues and BrC7K were introduced into different positions of the PD-L1 ECD corresponding to the Azi cross-linked hits (Fig. 2c). Rational selection also considered the 3D structures of the PD-L1 ECD homodimer. Our experiments demonstrated that PD-L1-Y123C and PD-L1-Q66BrC7K formed cross-linked homodimers, confirming that PD-L1 asymmetrically homodimerized (Fig. 3f). Altogether, we confirmed that PD-L1 asymmetrically homodimerized through the TM domain, intracellular domain, and extracellular domain.

### Glycosylation is important for PD-L1 homodimerization

**Fig. 4:**
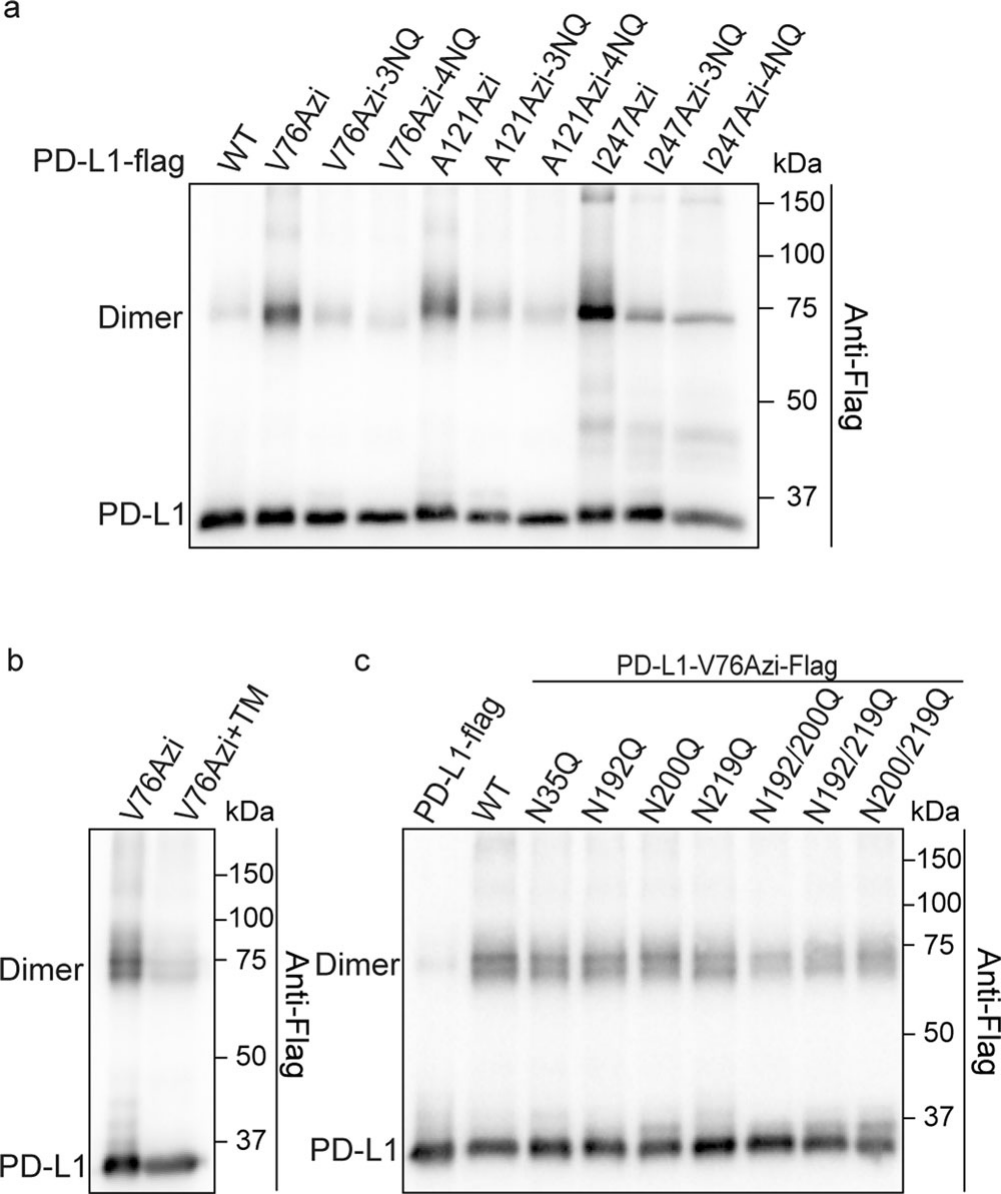
Glycosylation plays an important role in PD-L1 homodimerization. **a**, **b** Defect of PD-L1 glycosylation result in PD-L1 homodimerization attenuated. **a** PD-L1 and PD-L1-3NQ/4NQ mutants were incorporated with Azi. Azi introduced positions are indicated in the upper row. Cells were treated with UV for 20 min before collecting. **b** Tunicamycin (TM) suppresses PD-L1 homodimerization. Cells expressing PD-L1-V76Azi were treated with 5 µM tunicamycin for 24 h before collection. **c** The number of glycosylated residues affects homodimerization of PD-L1. Azi was incorporated into V76 of WT PD-L1-Flag or PD-L1-Flag mutants. All samples were treated with PNGase F to remove the N-glycan.

PD-L1 was extensively glycosylated at four conserved asparagine (Asn, N) residues (N35, N192, N200, and N219). Functionally, glycosylation is known to stabilize PD-L1 by preventing its degradation and plays important roles in PD-L1-mediated immunosuppression by regulating the PD-L1/PD-1 interaction. In the PD-L1 4NQ mutant, the four Asn residues were replaced with glutamine (Gln, Q) to completely abrogate glycosylation. Interestingly, cross-linking of PD-L1 homodimers were obviously suppressed by the 4NQ mutation in PD-L1 (Fig. 4a). Treating cells with tunicamycin, an N-linked glycosylation inhibitor that blocks the initial steps of protein glycosylation, confirmed that glycosylation plays important roles in PD-L1 homodimerization (Fig. 4b). To evaluate the glycosylation sites in PD-L1 that affect homodimerization, we constructed different PD-L1 variants, each of which carried one or two of the glycosylation site mutations and analyzed their effects on PD-L1 homodimerization. The results indicated that the number of glycosylated residues, but not the position of the glycosylation sites, contributed to the regulation of PD-L1 homodimerization (Fig. 4c).

### Homodimerized ITDs play critical roles in PD-L1 complex glycosylation

**Fig. 5:**
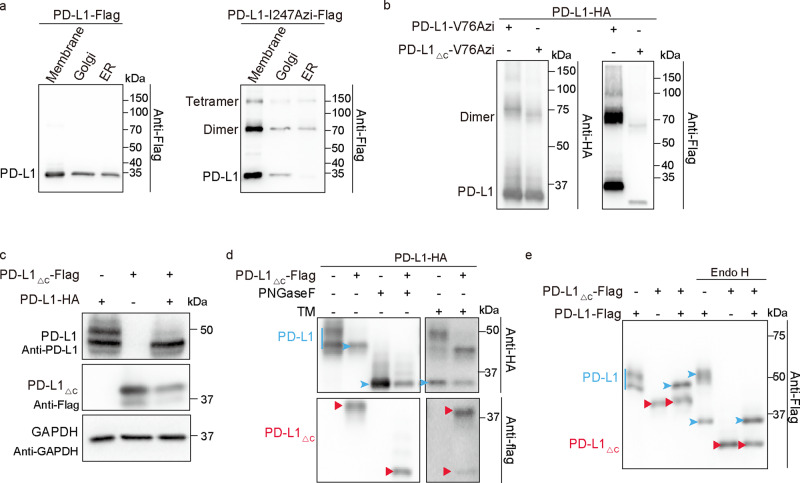
Homodimerized intracellular domains regulate PD-L1 complex glycosylation. **a** PD-L1 forms more homodimers in the Endoplasmic reticulum. HEK293T cells were transiently transfected with PD-L1-I247tag-Flag and pIRE4-Azi plasmids. 1 mM of Azi was added to the medium. Cells were treated with UV for 15 min. The ER, Golgi, and plasma membrane fractionation were isolated and separated on SDS-PAGE gels. Immunoblotting was performed using anti-Flag antibodies. **b** PD-L1_△C_ homodimerized with WT PD-L1. Azi was genetically incorporated into V76 of PD-L1-Flag or PD-L1_△C_-Flag in HEK293T cells expressing PD-L1-HA. Cells were treated with UV for 20 min. Cell lysates were treated with PNGase F to remove the N-glycan. **c**, **e** PD-L1_△C_ suppresses complex glycosylation of WT PD-L1. **c**, **d** WT PD-L1-HA and PD-L1_△C_-Flag plasmids were transfected into RKO KO PD-L1 Cells. **c** PD-L1_△C_ affects the pattern of PD-L1 that showed on immunoblotting. The anti-PD-L1 antibodies (clone number E1L3N) could recognize the full-length PD-L1, but not the PD-L1_△C_. **d** Cell lysates for lane 3 and lane 4 were treated with PNGase F. Samples for lane 5 and lane 6 were treated with 10 µM tunicamycin (TM) for 24 h before cell collecting. **e** RKO KO PD-L1 cells were cotransfected with PD-L1-Flag and PD-L1_△C_-Flag plasmids. Cell lysates for lanes 4–6 were treated with Endo H. Lines and arrowheads colored blue indicate full-length PD-L1. Red arrowheads indicate the PD-L1_△C_ proteins.

PD-L1 is anchored to the membrane by a hydrophobic transmembrane sequence, which is followed by a short intracytoplasmic region with very low sequence similarity to that of other B7 molecules. We isolated the ER, Golgi, and plasma membrane fractionation from cells expressing PD-L1-I247Azi and PD-L1. The ratio of monomeric to dimeric forms of PD-L1-I247Azi in the Golgi and plasma membrane is similar, which is higher than that in ER (Fig. 5a). These results suggest that highly mannosylated PD-L1 tends to homodimerize, which may play an important role in following PD-L1 complex glycosylation. Therefore, to explore the physiological function of PD-L1, we tried to identify PD-L1 homodimerization defect mutants. Our data indicated that the TM domain in PD-L1 plays an important role in its homodimerization. However, introducing mutations into the TM domain of PD-L1 failed to abort PD-L1 homodimerization (Supplementary Fig. 4a). The PD-L1_△C_ mutant, in which the intracytoplasmic region (R262-T290) was deleted, homodimerized with itself and with WT PD-L1 (Supplementary Fig. 4b and Fig. 5b). Interestingly, the expression of PD-L1_△C_ resulted in a reduction of the 50 kDa PD-L1 and an increase in the prevalence of a low-molecular-weight form (43 kDa) of WT PD-L1 (Fig. 5c). The heterogeneous expression pattern of PD-L1 shown by Western blot analysis suggested extensive N-glycosylation of four conserved asparagine residues. To determine whether the PD-L1_△C_-induced alteration of the PD-L1 pattern was caused by abnormal glycosylation, we treated samples with tunicamycin or the peptide-N-glycosidase (PNGase F), which removes all types of N-glycans (including complex glycans). Western blot analysis showed that both tunicamycin and PNGase F treatment resulted in a shift of all WT PD-L1 bands to reflect a molecular weight of ~34 kDa (non-glycosylated PD-L1 form) (Fig. 5d), suggesting that PD-L1_△C_ partially inhibited the N-glycosylation of WT PD-L1. We also treated cell lysates with Endo H, which cleaves only high-mannosylated and some hybrid N-glycans on N-linked glycoproteins. Basic N-Glycosylation that occurs in the endoplasmic reticulum (ER) is sensitive to Endo H digestion. Complex glycosylation, which has more than four different sugar types per glycan chain and occurs in the Golgi, are sensitive to PNGase F, but resistant to Endo H hydrolysis. The 43 kDa, but not the 50 kDa form of PD-L1 was converted into the 34 kDa form upon Endo H treatment (Fig. 5e), supporting the supposition that PD-L1_△C_ dimerization with WT PD-L1 suppressed its complex glycosylation. To identify the glycosylated residues of PD-L1 that were affected by PD-L1_△C_, we analyzed the effects of PD-L1_△C_ on the glycosylation of different PD-L1 variants. PD-L1_△C_ affected the glycosylation of all the PD-L1 mutants (Supplementary Fig. 4c). Therefore, the homodimerized ITD of PD-L1 plays a critical role in the PD-L1 complex glycosylation.

### Key arginine residues in the ITD are critical for complex PD-L1 glycosylation

We tried to map the key motifs/amino acids involved in regulating PD-L1 complex glycosylation. The small cytoplasmic domain of PD-L1 has been reported to regulate PD-L1 stability and function through multiple pathways. Previously, the “RMLDVEKC”, “DTSSK”, and “QFEET” were identified as possible conserved sequence motifs, and the “RMLDVEKC” and “DTSSK” motifs have been reported to regulate interferon-mediated cytotoxicity^48^. Acetylation of K263 in PD-L1 reportedly blocked PD-L1 nuclear translocation and caused reprogrammed expression of immune response-related genes^49^. The Xu laboratory revealed that huntingtin interacting protein 1 related (HIP1R) binds to the C-terminus of PD-L1 and delivers it to lysosomes for degradation^14^. Furthermore, the Xu and Hung laboratories separately demonstrated that palmitoylation at C272 of PD-L1 regulates its stability and trafficking^20, 22^. Therefore, we constructed different PD-L1 mutants in which these sequences were deleted or mutated amino acids, not in these motifs. Glycosylation of WT PD-L1 was not affected in cells expressing any of these mutants (Supplementary Fig. 5a–c). The interaction between HIP1R and WT PD-L1 was not affected by coexpression of the PD-L1_△C_ mutant (Supplementary Fig. 5d).

**Fig. 6:**
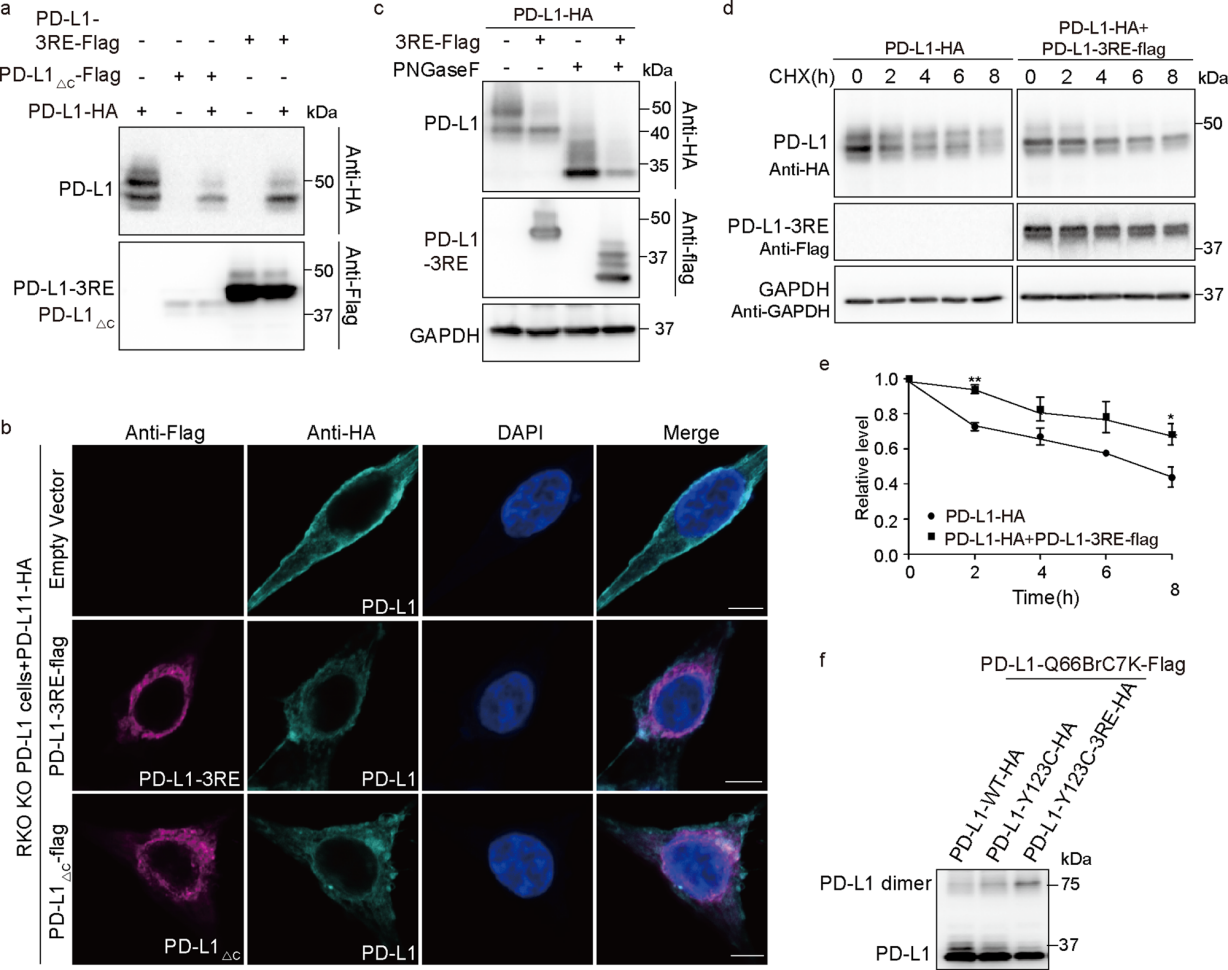
Dimerized key arginine residues in the intracellular domain play a critical role in PD-L1 complex glycosylation. **a** PD-L1-3RE mutant suppresses complex glycosylation of WT PD-L1. PD-L1-HA plasmid was cotransfected with PD-L1-3RE-Flag or PD-L1_△C_-Flag into RKO KO PD-L1 cells. **b** Plasma membrane localization of WT PD-L1 attenuates in cells expressing PD-L1-3RE mutant. Shown are immunofluorescence of cells expressing PD-L1-HA and PD-L1-3RE-Flag/PD-L1_△C_-Flag. Representative images are shown for each condition. Scale bars, 5 μm. **c** Total expression level of WT PD-L1 decreased in cells expressing PD-L1-3RE. RKO KO PD-L1 cells were transfected with PD-L1-HA or cotransfected with PD-L1-HA and PD-L1-3RE-Flag. Samples of lanes 3–4 were de-glycosylated with PNGase F. **d**, **e** PD-L1-3RE stabilized highly mannosylated PD-L1. PD-L1-HA or PD-L1-HA and PD-L1-3RE-Flag were transfected into RKO KO PD-L1 cells. The duration that cells treated with 150 µM CHX was labeled at the upper row. The quantification data represent the mean ± SEM for three independent experiments. *P ≤ 0.05, **P ≤ 0.01. **f** Effect of 3RE mutation on conformation of the PD-L1 ECD homodimer. Samples were treated with PNGase F to remove the N-glycan.

Recently, positive charges of PD-L1 R260/R262/R265 were found to be critical for the membrane association of PD-L1-ITD and for regulating PD-L1 stability^50^. Mutation of these basic residues (PD-L1-3RE mutant) impaired its membrane-binding capability and promoted its ubiquitination-dependent degradation. Therefore, we coexpressed the PD-L1-3RE mutant with WT PD-L1 and observed abnormal glycosylation of WT PD-L1 similar to that observed with the PD-L1_△C_ mutant, indicating that the three arginine residues in the ITD play critical roles in complex PD-L1 glycosylation (Fig. 6a). The membrane location of WT PD-L1 was also attenuated in cancer cells expressing the PD-L1-3RE mutant and PD-L1_△C_ mutant (Fig. 6b). Consistent with previous reports that 3RE mutations promoted PD-L1 degradation, the expression level of total WT PD-L1 decreased when it was coexpressed with the PD-L1-3RE mutant (Fig. 6c). Interestingly, the cycloheximide (CHX) chase assay revealed that degradation of the 43 kDa PD-L1 was slowed down in cells expressing PD-L1-3RE (Fig. 6d, e), indicating that the homodimerized ITD plays a negative role in stabilizing the 43 kDa PD-L1 protein.

We hypothesized that homodimerized ITD may facilitate PD-L1 transport from the endoplasmic reticulum to the Golgi apparatus, which is required for complex glycosylation. However, immunofluorescence staining showed that PD-L1-3RE coexpression resulted in WT PD-L1 accumulation in the Golgi, but not in the ER (Supplementary Fig. 6a, b). These results indicate that ITD homodimerization plays an important role in PD-L1 transport from the Golgi to the plasma membrane. Another hypothesis is that the homodimerized C-terminus impacts the ECD structure, which could attenuate PD-L1 complex glycosylation. To test this possibility, we tested the intensity of chemical cross-linking between PD-L1-123C-3RE and PD-L1-66Brc7K. Compared with WT PD-L1-123C, the PD-L1-123C-3RE mutant cross-linking with PD-L1-66Brc7K dramatically increased (Fig. 6f), which suggests that the 3RE mutation may cause conformational rearrangements of the PD-L1 ECD homodimer.

### Incomplete glycosylation of PD-L1 attenuates PD-1 binding and enhances T-cell toxicity

The previously published crystal structure of PD-1/PD-L1 showed that the ECD of PD-1 binds with the BMS-induced PD-L1 homodimerization surface^51^. We demonstrated that the PD-L1/PD-1 interaction surface is involved in physiological PD-L1 homodimerization (Fig. 2b, c). The partial overlap of the PD-L1 homodimerization interface with the PD-L1/PD-1 interaction interface indicates that PD-1 binding may influence PD-L1 homodimerization. In the presence of PD-1, the PD-L1 Y56/V76/Y123Azi mutants were covalently cross-linked with the extracellular domain of PD-1 (L25-Q167) (Supplementary Fig. 7a–c), while the other mutants (e.g., Q47Azi) failed to capture PD-1/PD-L1 binding. The homodimer cross-linking efficiency of these PD-L1-Azi mutants was partially suppressed in the presence of PD-1 (Supplementary Fig. 7d).

**Fig. 7:**
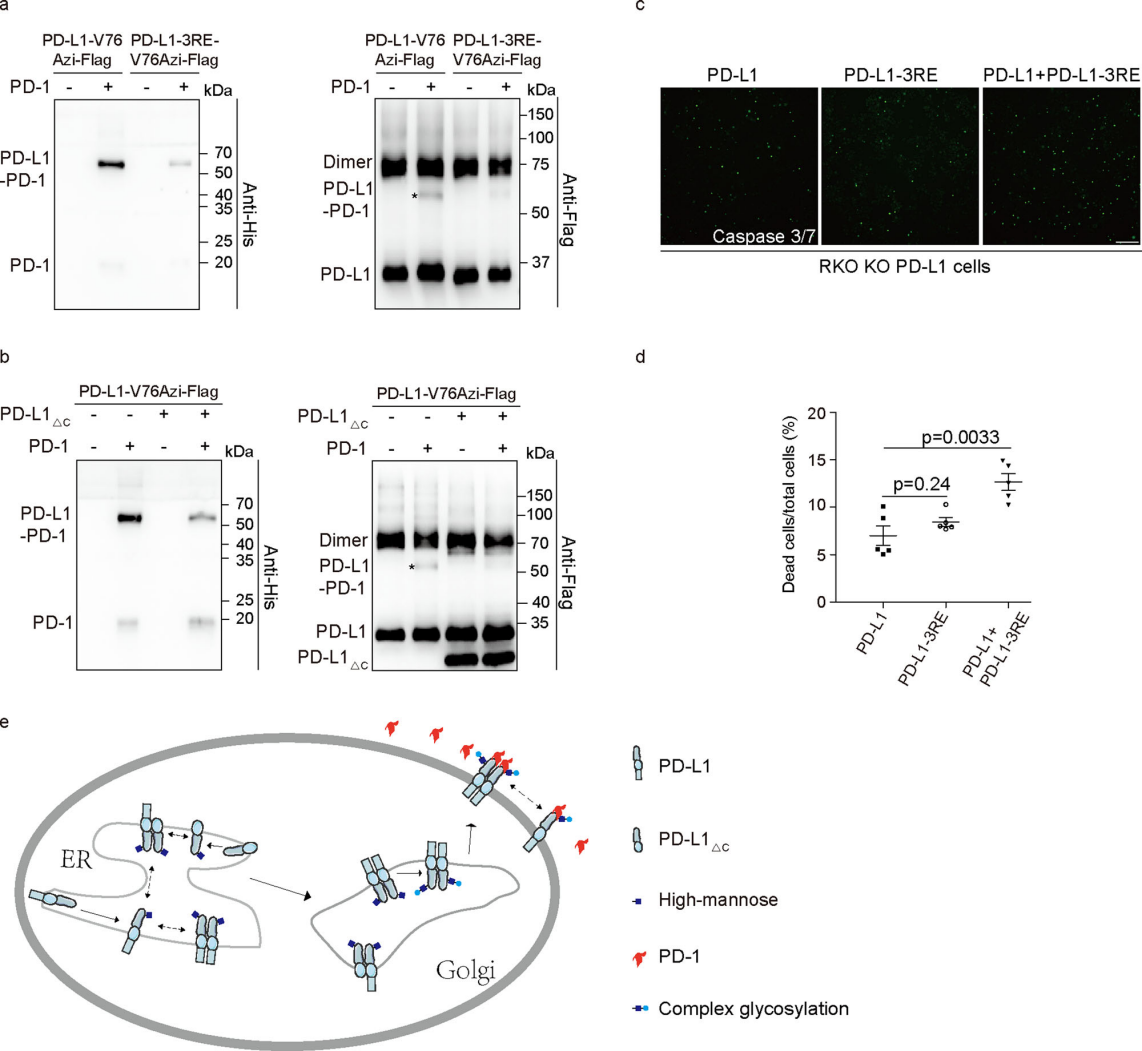
PD-L1 homodimerization plays an important role in PD-1 binding and T-cell toxicity. **a** PD-L1-Azi interaction with PD-1 was suppressed by 3RE mutation. Azi was incorporated at V76 of WT PD-L1 or PD-L1-3RE mutant. HEK293T cells were irradiated with UV in the absence or presence of PD-1-His protein. Shown are immunoblotting using anti-Flag and anti-His antibodies. **b** PD-L1_△C_ suppressed WT PD-L1 cross-linking with PD-1. Cells expressing PD-L1-V76Azi-Flag or coexpressing PD-L1-V76Azi-Flag and PD-L1_△C_-Flag were incubated with PD-1-His protein. Covalent cross-linking was induced by UV treatment for 20 min. **c**, **d** T-cell mediated tumor cell killing assay in RKO KO PD-L1 cells expressing PD-L1 and PD-L1-3RE. Green fluorescent was counted as dead cells. Representative phases are shown. Scale bar, 100 μm. **d** The ratio of dead cells was quantified. *N* = 5. **e** Schematic diagram of PD-L1 homodimer function. All samples were treated with PNGase F to remove the N-glycan.

The glycosylation of PD-L1 has been reported to regulate the PD-L1/PD-1 interaction. As expected, the loss of PD-L1 glycosylation (4NQ and 3NQ mutants) dramatically reduced the PD-1/PD-L1 interaction (Supplementary Fig. 7d). We also tested PD-L1-Azi cross-linking with PD-1 in the presence of the PD-L1-3RE mutant, which has been shown to suppress complex glycosylation of WT PD-L1. Our cross-linking experiments showed that the PD-L1-3RE mutant indeed suppressed WT PD-L1-Azi cross-linking with PD-1 (Fig. 7a). Consistently, PD-L1_△C_, which affects WT PD-L1 complex glycosylation, also suppressed WT PD-L1 cross-linking with PD-1 (Fig. 7b). Consistently, the cells coexpressing PD-L1-3RE and WT PD-L1 are more sensitive to human T-cell mediated cytolysis than RKO KO PD-L1 cells expressing WT PD-L1 or PD-L1-3RE mutant (Fig. 7c, d). These results together supported that homodimer-mediated complex glycosylation of the PD-L1 is important for PD-1 binding (Fig. 7e) and provides an advantage for the immune escape of tumor cells.

## Discussion

As a major target for immunotherapy, PD-L1 regulation has been extensively studied. However, the physiological state of PD-L1 (monomeric or dimeric) and the function of the PD-L1 homodimer are not clear. B7 family members have been reported to behave as dimers and monomers. Although the tendency of B7 proteins to dimerize is weak, their dimerization which may facilitate oligomerization, has been suggested to be favored on the cell surface^52^. In addition, the B7 proteins recruited by their ligands could increase their local concentration, which constrains them in an orientation more favorable for dimerization^52^. Despite evidence showing weak PD-L1 ECD homodimerization and drug-induced PD-L1 ECD homodimerization in vitro, full-length PD-L1 homodimerization under physiological conditions still waits to be explored. In this study, we demonstrated that membrane-anchored full-length PD-L1 forms homodimers in living cells. We further found that natural homodimers are formed through the TM domain, the ECD, and the ITD. Surprisingly, we discovered that Azi-replaced residues on the interaction interface of BMS-induced homodimer exhibit stronger cross-linking. This may be caused by glycosylation on N35, which is located at the surface-mediated nature PD-L1 homodimerization. Notably, homodimers were formed only when BrC7K, but not BetY and 4-Azido-L-phenylalanine, was incorporated into the ITD of PD-L1. The phenylalanine-based UAAs (4-Azido-L-phenylalanine and BetY) have rigid and short side chains, while the lysine-based BrC7K has longer and flexible linkers. This may explain our results that lysine-based BrC7K, but not the phenylalanine-based UAAs, captures the ITD-mediated PD-L1 dimer. These results indicated that PD-L1-BrC7K captures homodimer is specific.

The biological functions of naturally formed PD-L1 homodimers remain unclear. Our study found that highly mannosylated PD-L1 tends to homodimerize, while further PD-L1 modification in Golgi lowers the proportion of PD-L1 dimer. Consistently, the PD-L1 dimerization defect resulted in blockage of the partially glycosylated PD-L1 (43 kDa, high mannose) from being further glycosylated into the complex type (~50 kDa). These results suggested that homodimerization may play an important role in PD-L1 complex glycosylation. However, the distinct physiological function of monomeric and dimeric forms of PD-L1 remains further elucidation. In addition, we demonstrated that PD-L1-3RE expression resulted in WT PD-L1 accumulation at the Golgi apparatus. Importantly, proper PD-L1 glycosylation and transportation to the plasma membrane are critical for PD-L1 biological function. In particular, R260/R262/R265 in PD-L1 was found to play a critical role in its complex glycosylation. PD-L1 mutations at R260 and R262 were found in somatic cells of humans with cancers^48^. The PD-L1 mutants could homodimerize with WT PD-L1, and dominantly affect its complex glycosylation and plasma membrane localization.

The regulation of PD-L1 glycosylation has important clinical implications^13, 29, 30^. N-linked glycosylation has been reported to maintain PD-L1 stability and PD-L1 interaction with PD-1 to promote cancer cell evasion from T cells^18^. In addition, based on the structures of hPD-L1/mouse PD-1 (mPD-1) and mPD-L2/mPD-1, PD-1 has been proposed to bind dimeric PD-L1^17^. Moreover, compared to the PD-L1 monomer, artificially tetramerized PD-L1 has been reported to bind PD-1 with higher affinity^53^. Thus, the attenuated PD-L1/PD-1 binding ability may be caused by PD-L1 homodimerization defects and related abnormal glycosylation. We proved that complex glycosylated PD-L1, but not highly mannosylated PD-L1, is preferred substrate for PD-1. Therefore, abnormal glycosylation of PD-L1, which is caused by homodimerization defects contributes to attenuate PD-L1 binding with PD-1 and triggers T-cell-induced toxicity. However, deletion of the intracellular domain did not affect PD-L1 homodimerization, and we cannot rule out the possibility that PD-L1 dimerization facilitates PD-L1 binding with PD-1. Mutants or molecules that abrogate PD-L1 homodimerization without affecting PD-L1 glycosylation would be useful in clarifying this mechanism.

Finally, our study, together with other studies, indicated that the cytoplasmic domain of PD-L1 regulates PD-L1 glycosylation, degradation, binding with PD-1, and so on through various mechanisms, making it an attractive target for immunotherapy and blockade of PD-L1-related cancer signaling. Peptides targeted to PD-L1-CD were found to reduce PD-L1 levels by enhancing PD-L1 lysosomal degradation^14^ or blocking its palmitoylation^22^. Overall, our study will help to identify and develop novel inhibitors of PD-L1/PD-1 signaling to combat tumor immunosuppression.

## Methods

### Antibodies and reagents

The sources of antibodies are listed as follows: rabbit anti-PD-L1 (13684, CST; WB 1:2000), rabbit anti-HA (ab9110, Abcam; IF 1:400), mouse anti-HA (M20003, Abmart; WB 1:2000), mouse anti-flag (M20008, Abmart; IF 1:400, WB 1:2000), rabbit anti-Strep II (HA500061, HuaBio; WB 1:2000), mouse anti-GAPDH (AC002, ABclonal; WB 1:4000), mouse anti-PDI (MA3-019, Thermofisher; IF 1:100), mouse anti-GM130 (610822, BD; IF 1:100), HRP-conjugated goat anti-rabbit IgG (H + L) (SA00001-2, Proteintech; WB 1:2000), HRP-conjugated goat anti-mouse IgG (H + L) (31430, Invitrogen; WB 1:2000), goat anti-Rabbit IgG(H + L) cross-adsorbed Alexa Fluor 488 (A-11008, Thermofisher; IF 1:400), goat anti-mouse IgG(H + L) highly cross-adsorbed Alexa Fluor Plus 647 (A32728, Thermofisher; IF 1:400). The reagents are listed as follows: BMS-1166 (S8859, Selleck), CHX (S7418, Selleck), Tunicamycin (HY-A0098, MCE), PD-1-His protein (ab174035, Abcam), PNGase F (P0704, NEB), Endo H (P0703, NEB), 4-Azido-l-phenylalanine (33173-53-4, SustGreen Tech), BetY (481052-60-2, SustGreen Tech), BrC7K (2211978-09-3, SustGreen Tech), puromycin (HY-B1743A, MCE), KOD DNA polymerase (KMM-101, TOYOBO), protease inhibitor cocktail (HY-K0010, MCE), anti-Strep II affinity beads (NRPB61, NUPTEC), ECL western blotting substrate (1705061, Bio Rad), RIPA lysis buffer (HY-K1001, MCE), lipofectamine 2000 (11668019, Thermofisher), PEI (24765-2, Polysciences), ER extraction kit (EX1370, Solarbio), and Golgi protein extraction kit (EX1240, Solarbio).

### Plasmids

The mammalian expression vector for PD-L1 was synthesized by GENERAL BIOSYSTEMS. The pIRE4-Azi plasmid was purchased from Addgene. The pHY-XYRS plasmid was constructed and used in our previous work^47^. The pHY-BrC7KRS plasmid was derived from pHY-XYRS, and contains four copies of the suppressor tRNA driven by U6 promoter and one copy of BrC7KRS driven by a CMV promoter. cDNA for HIP1R was amplified from HEK293T cDNA library. Genes were inserted into pcDNA3.1(+) or pCMV-HA/Flag for mammalian expression. To generate PX330-sgPD-L1, the sequence of guide RNA (gRNA) used for PD-L1 is 5’-ATTTACTGTCACGGTT-CCCA-3’. To generate PUC19-PD-L1, homologous arms were amplified using the following primers: for left, forward 5’-TTAAAATGCATGCAATTTTCTTATAGAG-3’ and reverse 5’-TTCCTCGAAAC-ATTTACAAAATTGG-3’, for right, forward 5’-TTGAATGCAAATTCCCAGTAG-3’ and reverse 5’-GGTTTGTTCTGAATATATTTATTTGC-3’. PD-L1 mutants were generated by site-directed mutagenesis or homologous recombination. All plasmids were sequenced to confirm whether the designed mutations were present, without any other unwanted mutation.

### Cell culture and transfection

HEK293T cells were purchased from National Collection of Authenticated Cell Cultures, RKO cells were purchased from iCell Bioscience Inc. All cell lines were tested negative for mycoplasma contamination. The above cells were maintained in DMEM medium supplemented with 10% FBS and cultured in a humidified incubator at 37 °C under 5% CO_2_. Transient transfection was performed using Lipofectamine 2000 or PEI, following the manufacturer’s instructions.

### CRISPR/Cas9 knockout cell lines

Construction of knockout cells by CRISPR/Cas9 was performed as described^54^. Knockout cells were constructed by PX330-sgPD-L1 and PUC19-PD-L1 plasmids. The PX330-sgPD-L1 and PUC19-PD-L1 vectors were cotransfected into RKO cells. Forty-eight hours post transfection, cells were sub-cultured into complete DMEM with 2 mg/mL puromycin. Two weeks later, cells were seeded into 96-well plates at super low density to screen single colonies. The expression of endogenous PD-L1 was tested by immunoblotting, and PD-L1 knockout clones were confirmed by DNA sequencing.

### Uaa incorporation and cross-linking

The pIRE4-Azi, or pHY-BrC7KRS or pHY-XYRS plasmid was cotransfected with the pcDNA3.1-PD-L1-Xtag-Flag vectors into cells for Azi, BrC7KRS, and XYRS incorporation individually. The final concentration of Uaas in culture medium was 1 mM for Azi, 0.5 mM for BrC7K, and 0.5 mM for BetY. Forty-eight hours post transfection, cells were harvested. Samples with Azi incorporation were irradiated with a UVP cross-linker for 20 min on ice. The cells were lysed in relevant lysis buffer and analyzed by immunoblotting.

### PD-L1 and PD-1 cross-linking assay

For PD-1 and PD-L1 protein interaction analysis, cells expressing PD-L1-XAzi were harvested and washed with PBS three times, then incubated with 20 µg/mL PD-1-His protein in PBS at room temperature for 30 min. The samples were irradiated with a UVP cross-linker for 20 min on ice and washed with PBS three times followed by subsequent protein extraction.

### Protein extraction

For total protein extraction, RIPA lysis buffer supplemented with 1% protease inhibitor cocktail was added to cells and lysed on ice for 10 min. After being cleared by centrifugation (16,000 × *g*) at 4 °C for 15 min, the supernatant was transferred into new tubes. Samples with SDS loading buffer were boiled at 95 °C for 5 min. Samples were treated with PNGase F or Endo H at 37 °C for 2 h to de-glycosylate the PD-L1 as indicated in the figure legends.

In order to obtain the cytoplasm protein and membrane protein individually, HDB buffer (140 mM NaCl, 5 mM KCl, 12.5 mM HEPES, 0.5 mM EDTA, 5 mM MgCl_2_, pH 7.4) supplemented with 1% protease inhibitor cocktail was added to cells. After repeating freeze–thaw cycles (37 °C water and liquid nitrogen) five times, samples were cleared by centrifugation (16,000 × *g*) at 4 °C for 15 min, and the supernatant was transferred into new tubes. Sediment was resuspended with membrane extraction buffer (1.5 mM NaCl, 50 mM HEPES, 1.5 mM MgCl_2_, 1 mM EGTA, 10% glycerol, 1% Triton X-100, 1% protease inhibitor cocktail, pH 7.5) and rotated at 4 °C for 30 min followed by 15 min 16,000×*g* centrifugation. The ER protein and Golgi protein extractions were performed using the ER extraction kit (EX1370, Solarbio) and the Golgi protein extraction kit (EX1240, Solarbio) individually, following the manufacturer’s instructions.

### Immunoprecipitation

For immunoprecipitation, cells were collected 48 h after transfection with appropriate plasmids and lysed in lysis buffer (50 mM Tris–HCl pH 8.0, 150 mM NaCl, 5 mM EDTA, and 0.5% NP-40) supplemented with 1% protease inhibitor cocktail for 30 min on ice. Cell lysates were centrifuged at 16,000×*g* for 15 min to remove the debris. Anti-Strep II affinity beads were added to the supernatant and incubated overnight at 4 °C with constant rotation. The beads were washed three times with lysis buffer and then boiled at 95 °C for 5 min.

### Immunoblotting analysis

For western blot analysis, samples were run on SDS-PAGE gels and transferred onto PVDF membranes. The membranes were blocked with 5% milk in TBST for 2 h and incubated with corresponding antibodies overnight at 4 °C, followed by three times TBST washing and incubating with HRP-conjugated secondary antibodies at room temperature for 2 h. The western blot bands were detected using an ECL western blotting substrate on a Sage Capture 0531 Imaging System.

### Immunofluorescence

For immunofluorescence, after washing with PBS, cells were fixed in 4% paraformaldehyde for 20 min and permeabilized with 0.25% TWEEN-20 in PBS at room temperature for 15 min. Samples were blocked with 10% normal goat serum in PBS at room temperature for 1 h, then sequentially stained with the indicated primary antibodies overnight at 4 °C and corresponding secondary antibodies at room temperature for 1 h in dark. The rest of the steps need to be done in dark. Coverslips were mounted onto slides with mounting medium containing DAPI. Images were acquired using a confocal laser scanning microscope (ZEISS LSM 880). Colocalization analysis was performed using ImageJ.

### T-cell cytotoxicity assay

Human peripheral blood mononuclear (PBMC) cells were isolated from the blood of a healthy donor. To acquire activated T cells, PBMCs were activated with 5 μg/mL CD3 antibody, 1 μg/mL CD28 antibody, and 10 ng/mL IL2 for 48 h. RKO KO PD-L1 cells expressing PD-L1, PD-L1 3RE or coexpressing PD-L1 and PD-L1 3RE, were cocultured with the activated T cells at a ratio of 1:5 for 24 h. Cell debris was removed by PBS wash, and cancer cells were incubated with fluorescence caspase-3/7 dye at 37 °C for 1 h. Cells were washed with culture medium and then incubated with Hoechst for 15 min. After washing twice with PBS, cells were observed under a fluorescence microscope.

### Statistical and reproducibility

We performed the analysis in GraphPad Prism 5, using the unpaired, two-tailed *t* test module. Statistical significance was considered when *P* value was below 0.05. Quantitative results are reported as the mean ± SEM.

### Reporting summary

Further information on research design is available in the Nature Research Reporting Summary linked to this article.

## Supplementary information

Supplementary Information

Description of Additional Supplementary Files

Supplementary Data 1

Reporting Summary

## Data Availability

All data supporting the findings of this study are available within the paper and the supplementary information. The source data behind the graphs can be found in Supplementary Data 1. Uncropped blots are available in Supplementary Fig. 8.
