# Supplementary material for: Homodimerized cytoplasmic domain of PD-L1 regulates its complex glycosylation in living cells
Source: Commun Biol. 2022 Aug 30;5:887. doi: 10.1038/s42003-022-03845-4 (PMC9427764; doi:10.1038/s42003-022-03845-4)

## Supplementary figures and legends

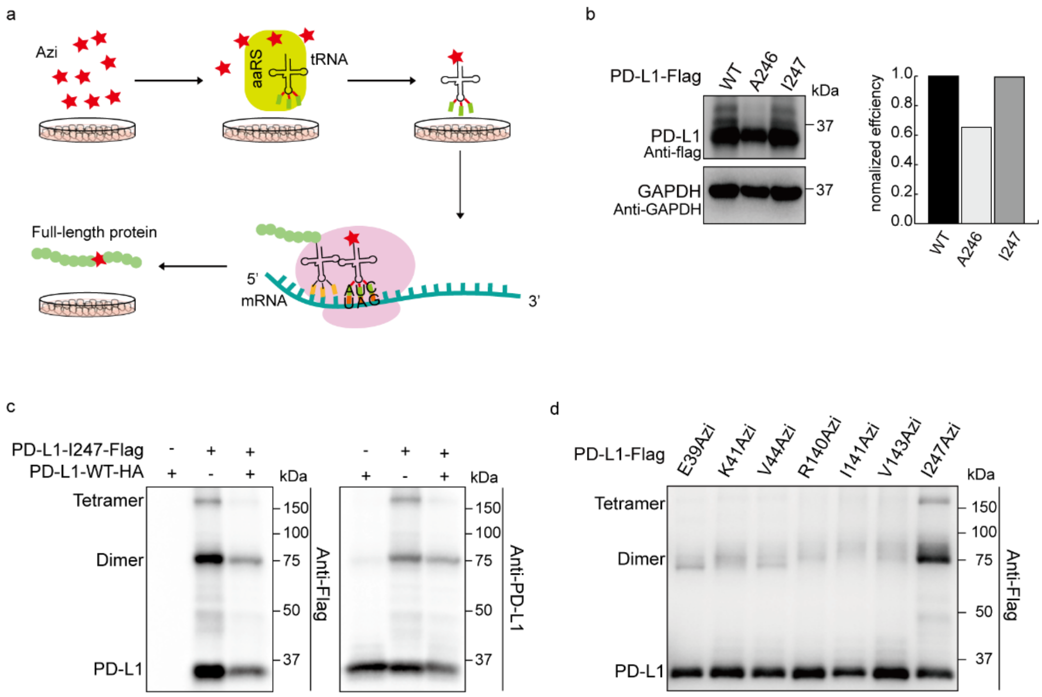


**Supplementary Figure. 1 Covalent capture of PD-L1 homodimer using genetically incorporated photocrosslinker**

(a) Schematic diagram of genetical incorporation of UAAs. (b) Replace specific residues of PD-L1 with Azi. PD-L1-Xtag-Flag and pIRE4-Azi plasmids were transfected into HEK293T cells for Azi incorporation. Azi introduced positions are indicated in the upper row. Azi incorporation efficiency was normalized to WT PD-L1. Samples were collected for anti-Flag and anti-GAPDH immunoblotting. (c) PD-L1 homodimers were detected with Anti-PD-L1 antibodies in UV-treated cells. (d) Azi covalent capture of PD-L1 homodimer is site specific. Azi incorporated positions are indicated in the upper row. Samples were treated with UV and collected for further immunoblotting analysis.

All samples were treated with PNGase F to remove the N-glycan.

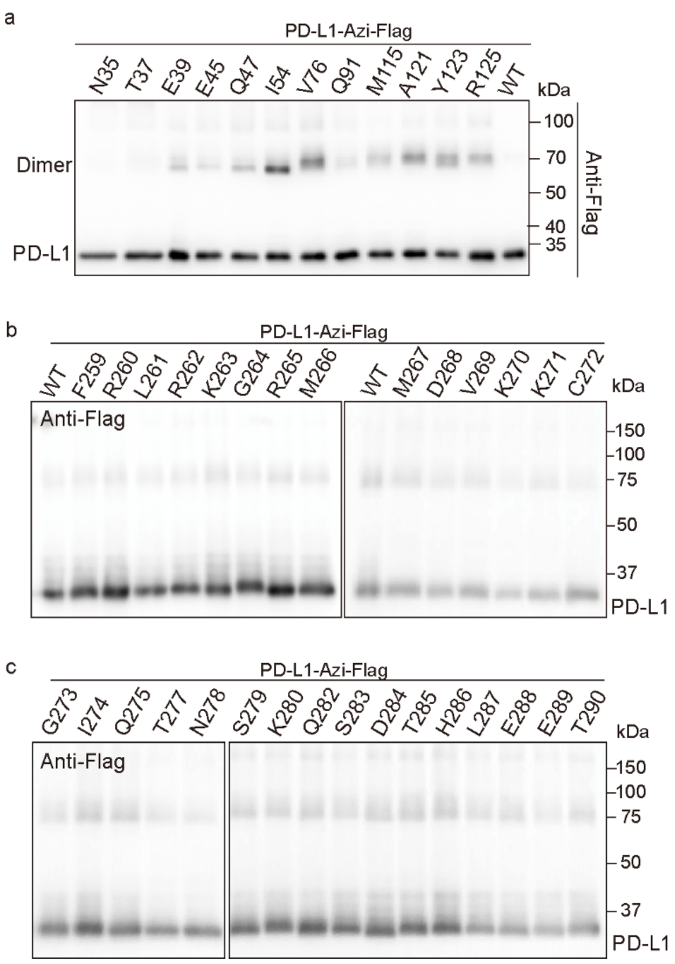


**Supplementary Figure. 2 Azi incorporated at the intracellular domain fail to capture PD-L1 homodimer**

(a) Azi amino acids replace residues on the interaction interface of the BMS-induced homodimer (I54, V76, M115, A121, Y123, and R125) show stronger crosslinking band than that of native homodimer (E39, E45, Q47, and Q91). Transfected cells expressing PD-L1-XAzi-Flag proteins were treated with UV to induce crosslinking and performed immunoblotting analysis. (b-c) PD-L1-Xtag-Flag and pIRE4-Azi plasmids were transiently transfected into HEK293T cells. 1 mM Azi was added to the cell culture medium. UV-treated cells were lysed and separated on SDS-PAGE gels, then analyzed with western blotting using anti-Flag antibodies. WT PD-L1 was used as control. Azi introduced positions in PD-L1 are indicated in the upper row.

All samples were treated with PNGase F to remove the N-glycan.

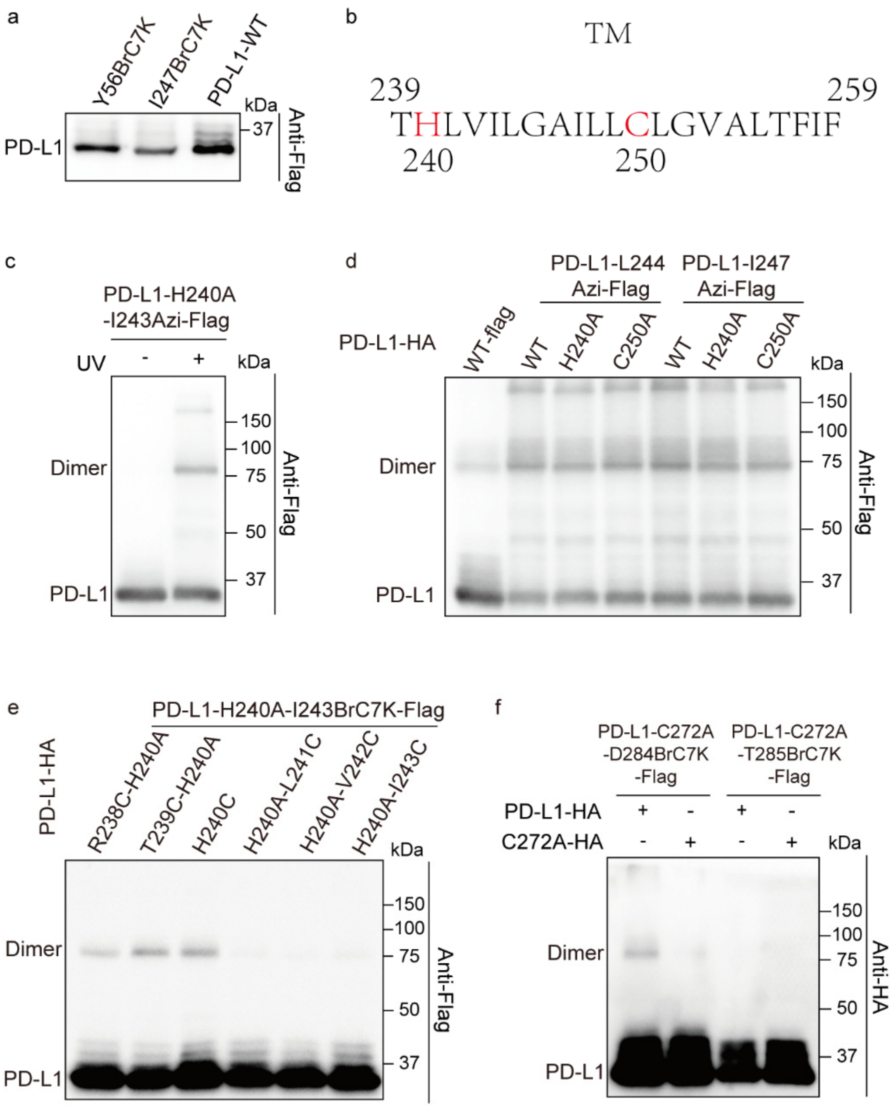


**Supplementary Figure. 3 BrC7K-mediated covalent capture of PD-L1 homodimer**

(a) Introducing of BrC7K into PD-L1 proteins. PD-L1-Xtag-Flag plasmids were cotransfected with pHY-BrC7KRS to incorporate BrC7K. Cells were cultured in medium with 0.5 mM BrC7K. (b) Amino acid sequence of the PD-L1 transmembrane domain. Potential targeted amino acids for BrC7K and BetY were colored red. (c-d) H240A and C250A mutations did not affect PD-L1 homodimerization. (c) PD-L1-H240A-I243Azi-Flag was expressed in HEK293T cells. (d) Cells coexpressing PD-L1-L244/I247Azi-Flag and PD-L1-H240A/C250A-HA mutant were treated with UV to induce covalent capture. (e) T239 and H240 of PD-L1 are interaction partners of I243. Cells were transfected with PD-L1-H240A-I243BrC7K-Flag and PD-L1-HA Cys mutants. (f) The intracellular domain is involved in PD-L1 homodimerization in living cells.

All samples were treated with PNGase F to remove the N-glycan.

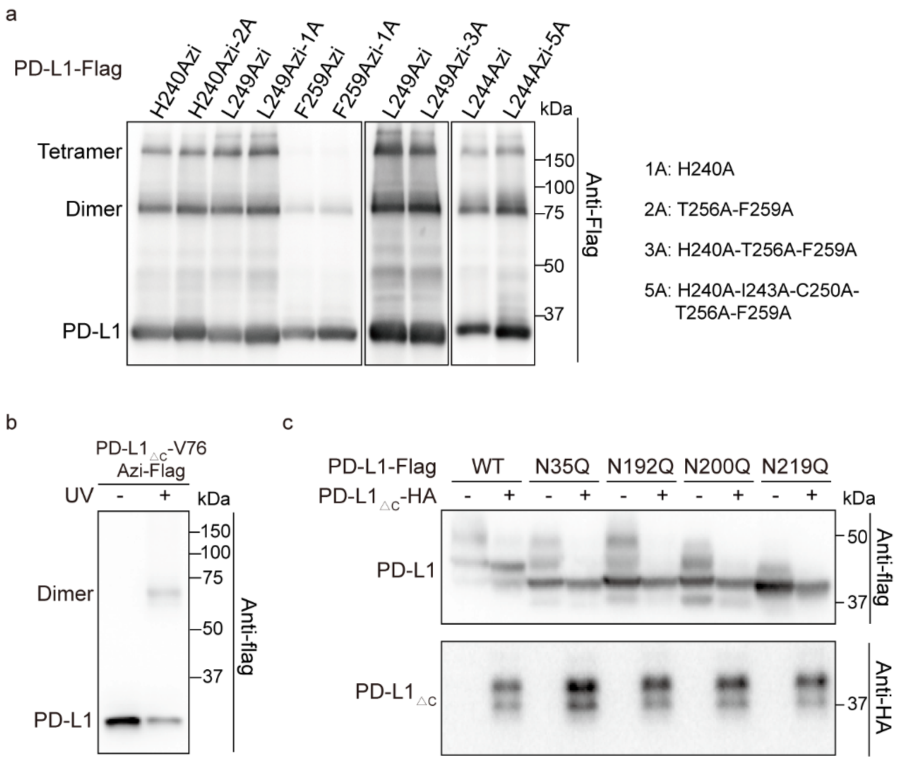


**Supplementary Figure. 4 Homodimerization of PD-L1 mutants**

(a) Mutations of transmembrane domain fail to destroy PD-L1 homodimerization. Samples were treated with PNGase F to remove the N-glycan. (b) Deletion the intracytoplasmic region of PD-L1 did not affect its homodimerized. Azi was incorporated into V76 of PD-L1$_{\triangle C}$ protein in HEK293T cells. Samples were treated with PNGase F to remove the N-glycan. (c) PD-L1$_{\triangle C}$ affected glycosylation of all Arg residues.

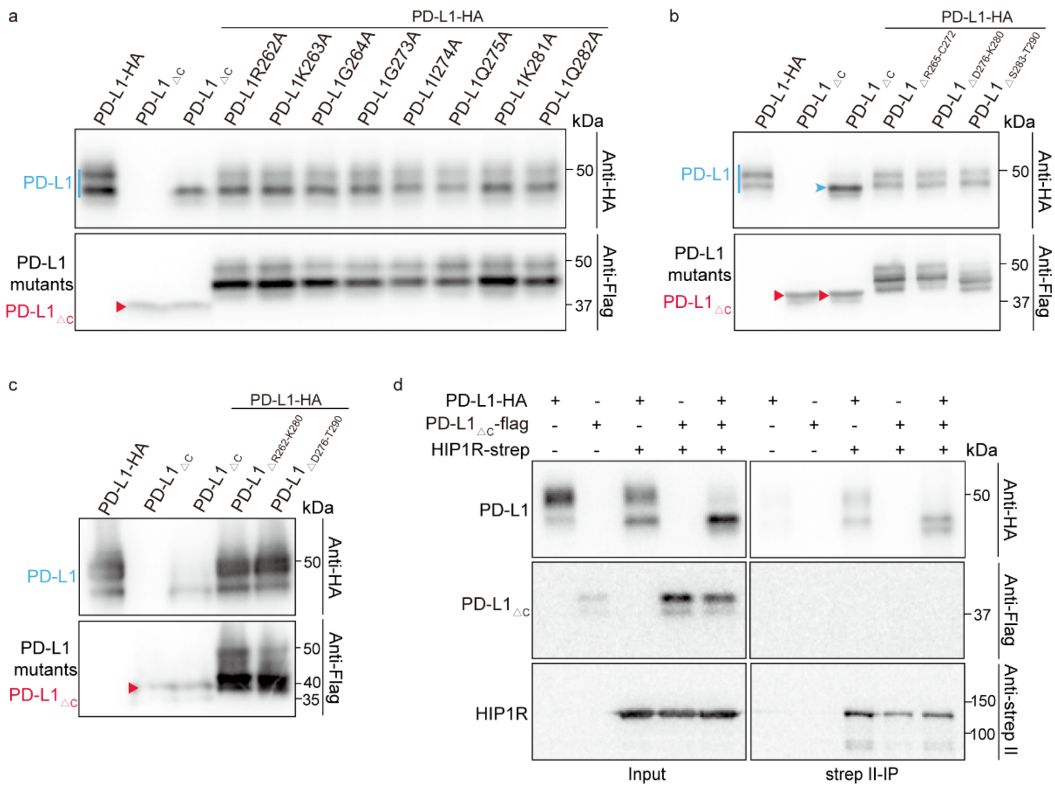


**Supplementary Figure. 5 Explore possible functions of various PD-L1 mutants**

(a-c) Effects of PD-L1 mutants on PD-L1 complex glycosylation. PD-L1-HA was cotransfected with various PD-L1-Flag mutants. Lines and arrowheads colored blue indicate full-length PD-L1. Red arrowheads indicate PD-L1$_{\triangle C}$ which were used as positive control. Transfected plasmids for each sample were labeled in the upper row. (d) Interaction between PD-L1 and HIP1R was not affected by PD-L1$_{\triangle C}$. Transfected plasmids for each lane were labeled in the upper row. Shown are immunoblots of Strep II IP and total cell lysates (Input).

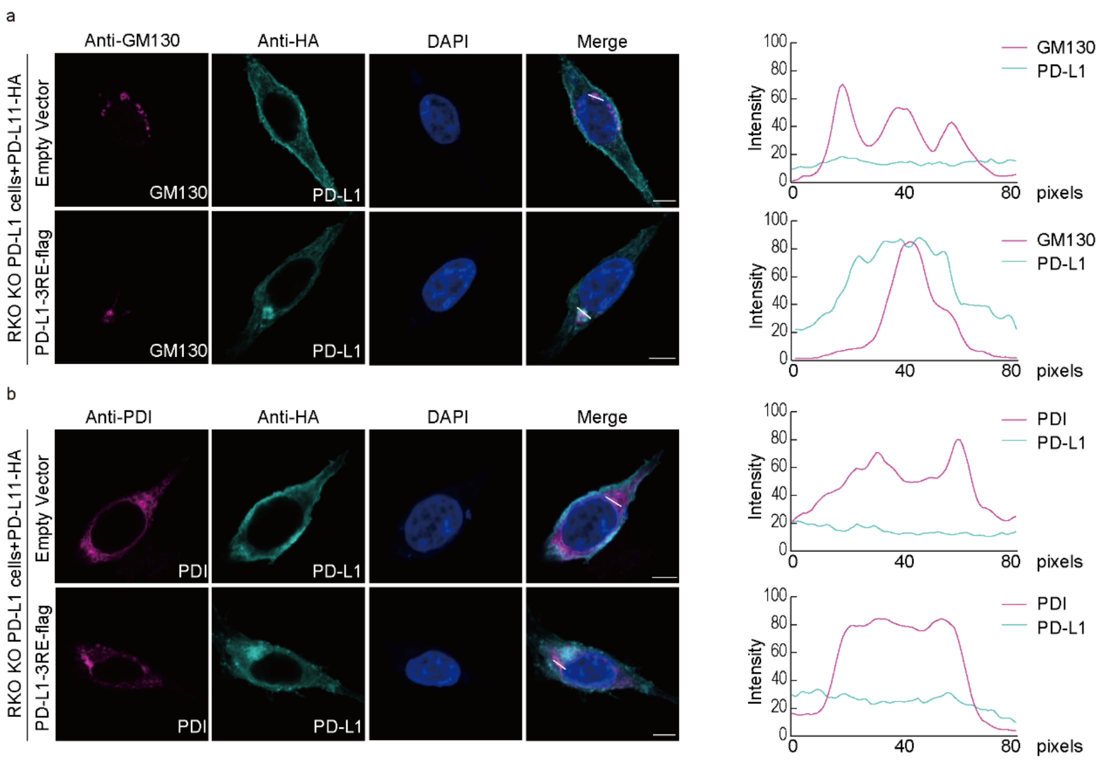


**Supplementary Figure. 6 Expression of PD-L1-3RE results in WT PD-L1 accumulating at the Gogi**

(a-b) PD-L1 colocalized with the Gogi, but not the ER in RKO KO PD-L1 cells expressing PD-L1-3RE. Cells expressing PD-L1-Flag or PD-L1-Flag and PD-L1-3RE-HA were stained with antibodies indicated in the figures. GM130 is the Gogi marker, PDI is the endoplasmic reticulum marker. Representative images are shown for each condition. The fluorescence intensity profiles of PD-L1 and markers along the white line are plotted on the right. Scale bars, 5 μm.

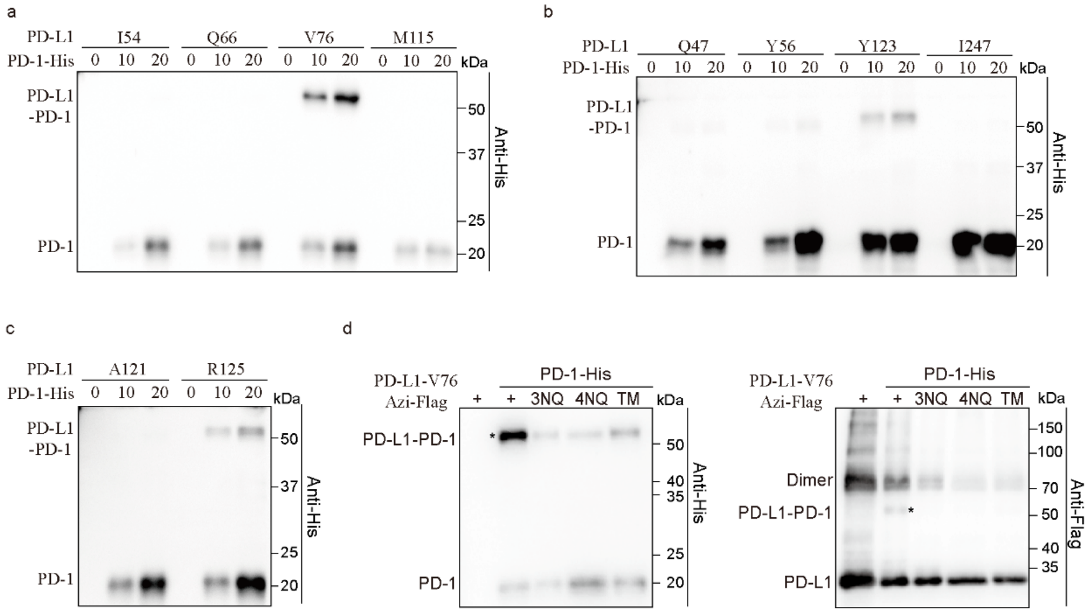


**Supplementary Figure. 7 PD-L1 covalently captures PD-1**

(a-c) V76, Y123, and R125 residues of PD-L1 are involved in interaction with PD-1. HEK293T cells expressing PD-L1-XAzi mutants were incubated with PD-1-His protein and irradiated with UV. Shown are immunoblotting using anti-His antibodies. (d) PD-L1 glycosylation plays an important role in PD-1/PD-L1 interaction. PD-1 partially suppresses PD-L1-V76Azi-mediated crosslinking of homodimer. 20 μg/mL PD-1-His protein were added to HEK293T cells expressing PD-L1-V76Azi mutants. UV irradiation was performed to induce crosslinking. The stars indicate the PD-L1-PD-1 covalent complexes.

All samples were treated with PNGase F to remove the N-glycan.

**Supplementary Figure. 8 Uncropped original western blots**

Fig.1c

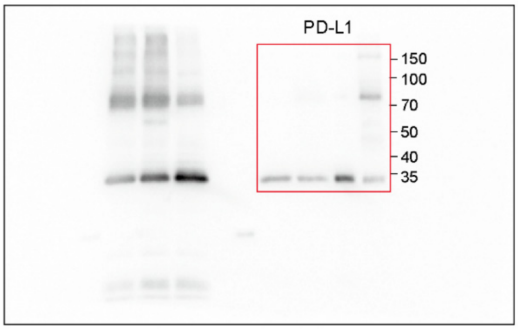


Fig.1d

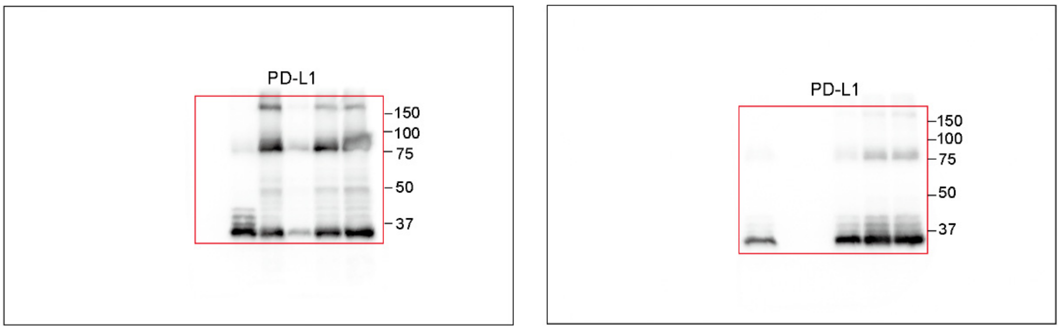


Fig.2a

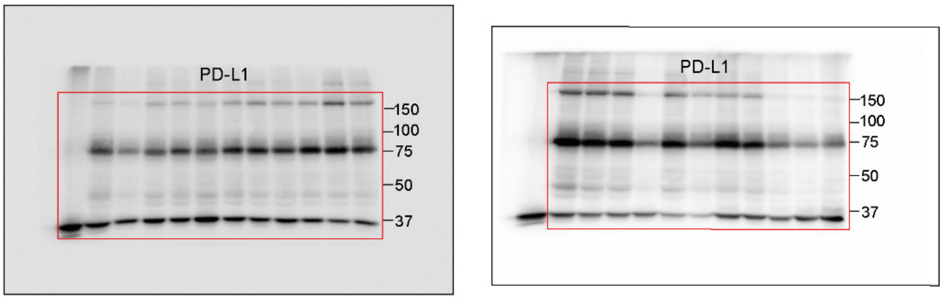


Fig.2c

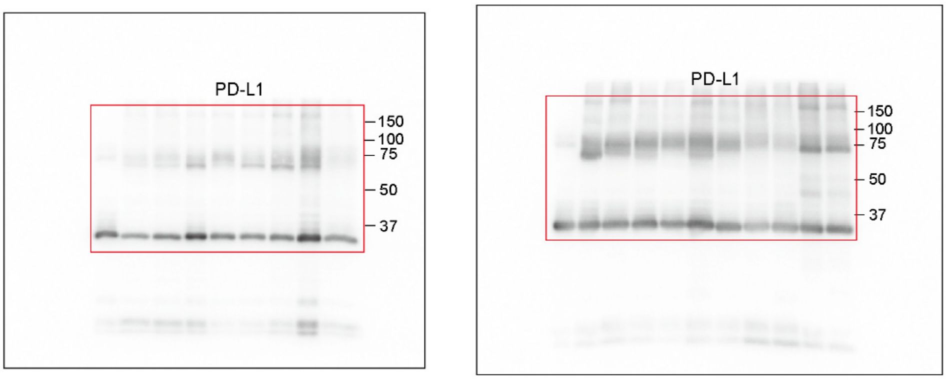

Fig.2d

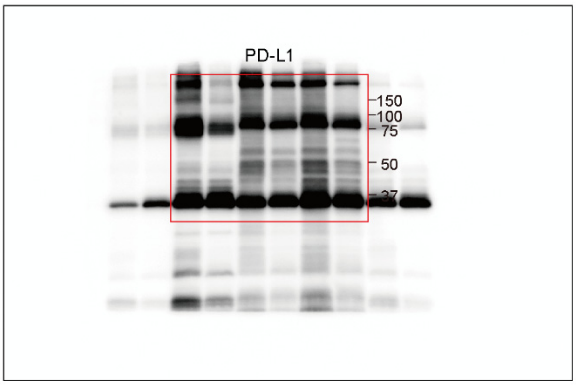


Fig.2e

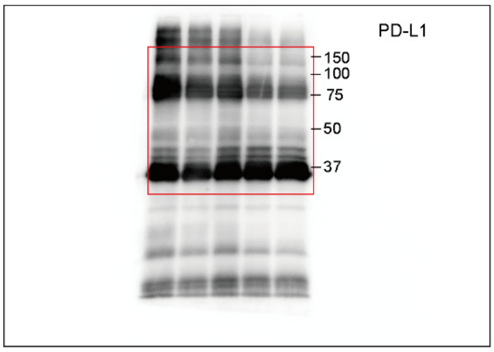


Fig.3c

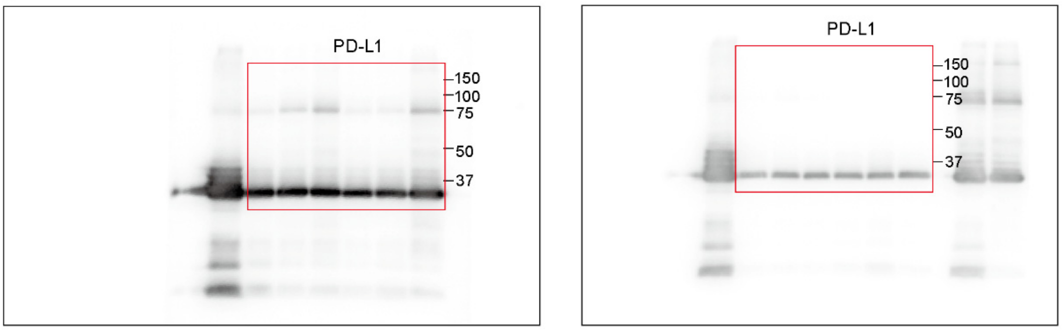


Fig.3d

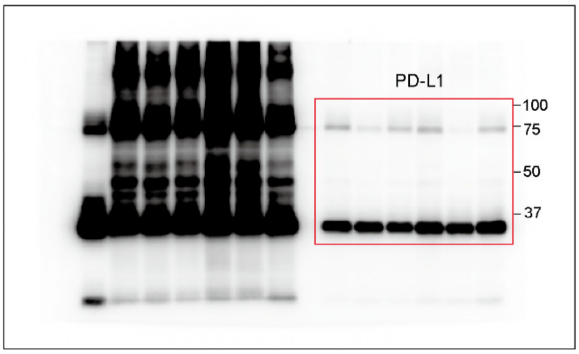


Fig.3e

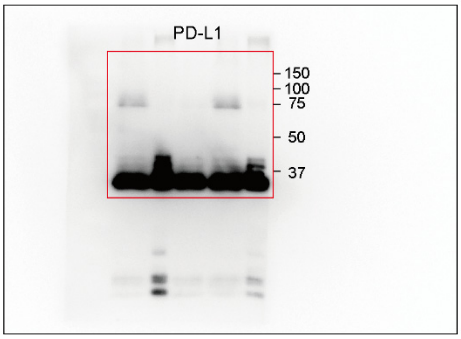


Fig.3f

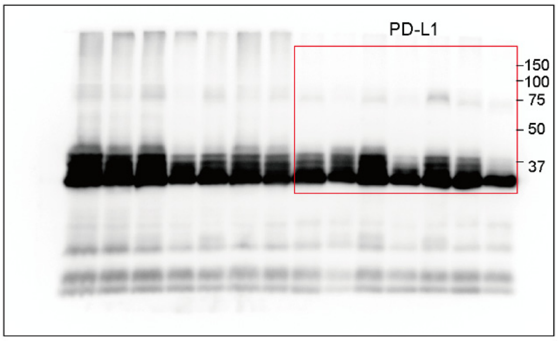

Fig.4a

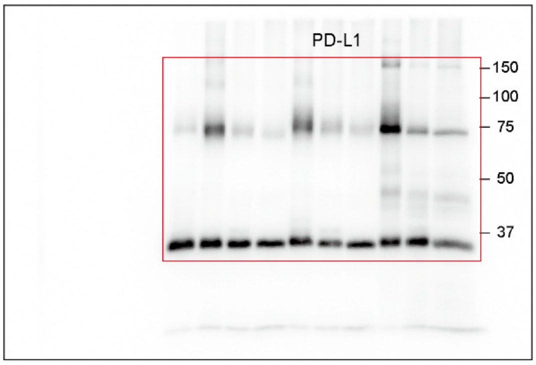


Fig.4b

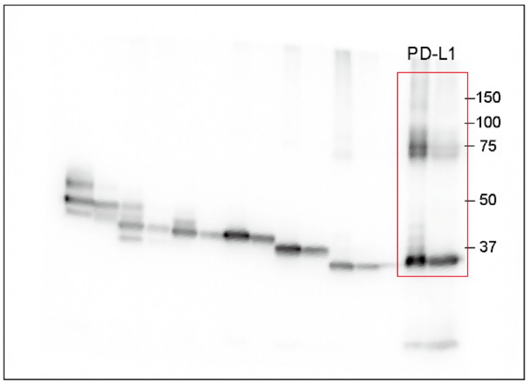


Fig.4c

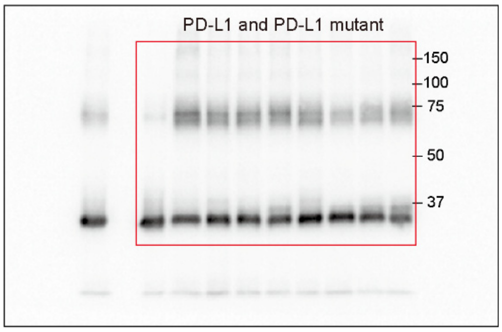


Fig.5a

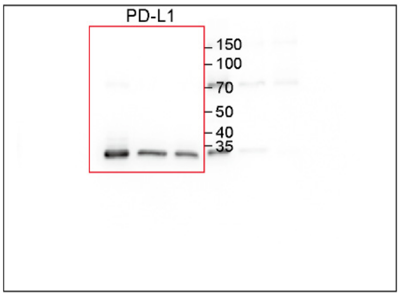


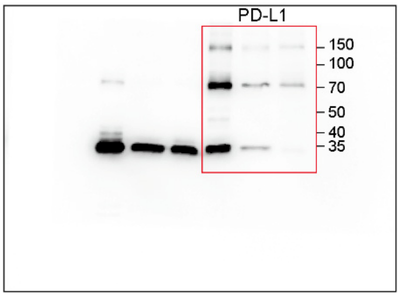


Fig.5b

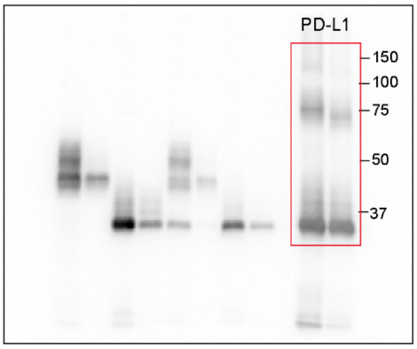


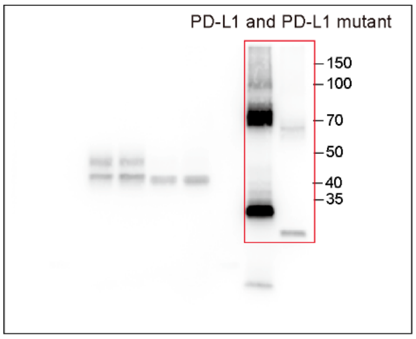

Fig.5c

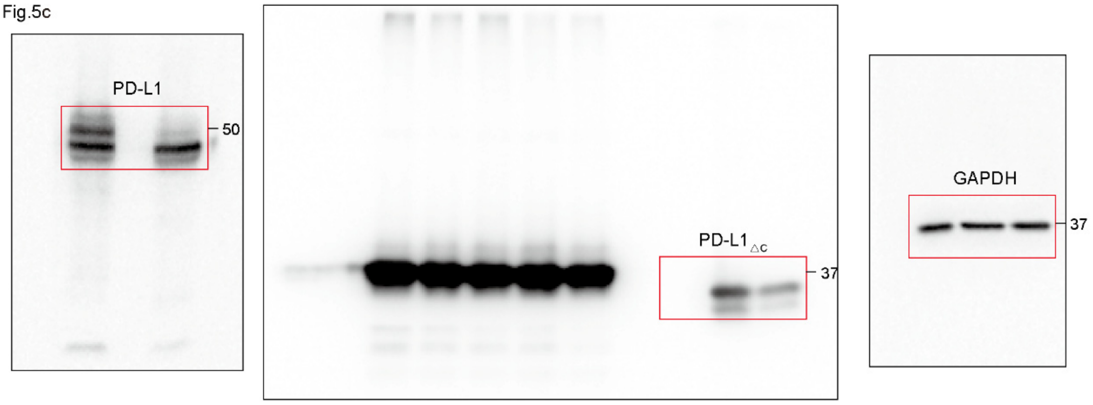


Fig.5d

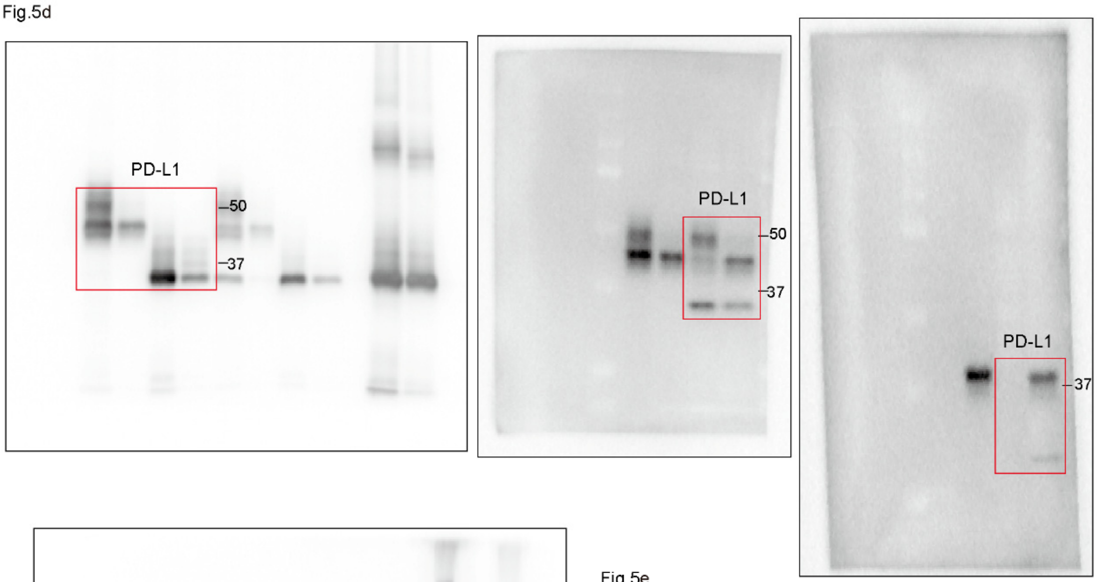


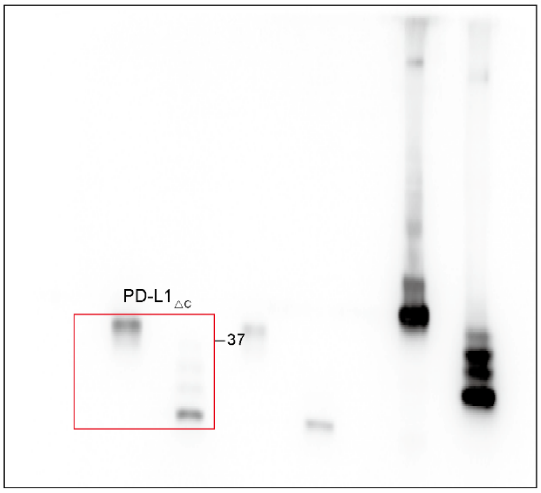


Fig.5e

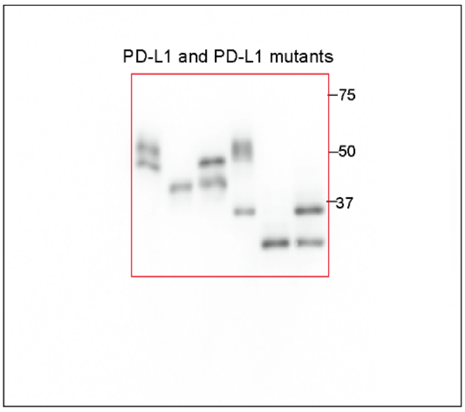

Fig.6a

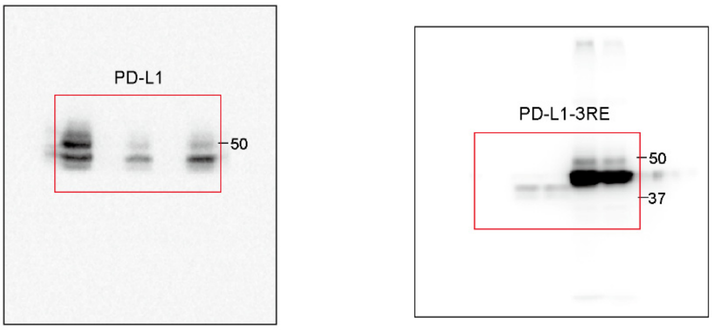


Fig.6c

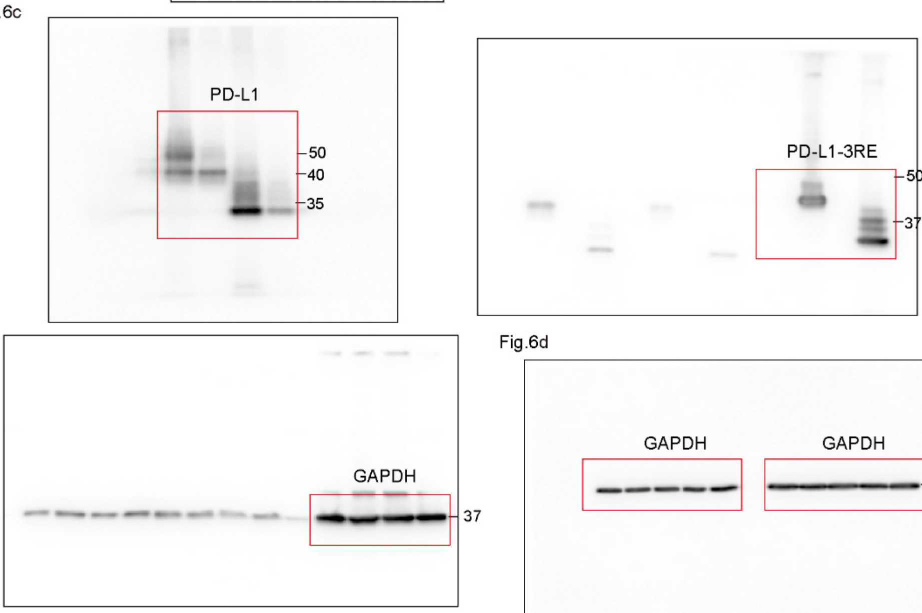


Fig.6d

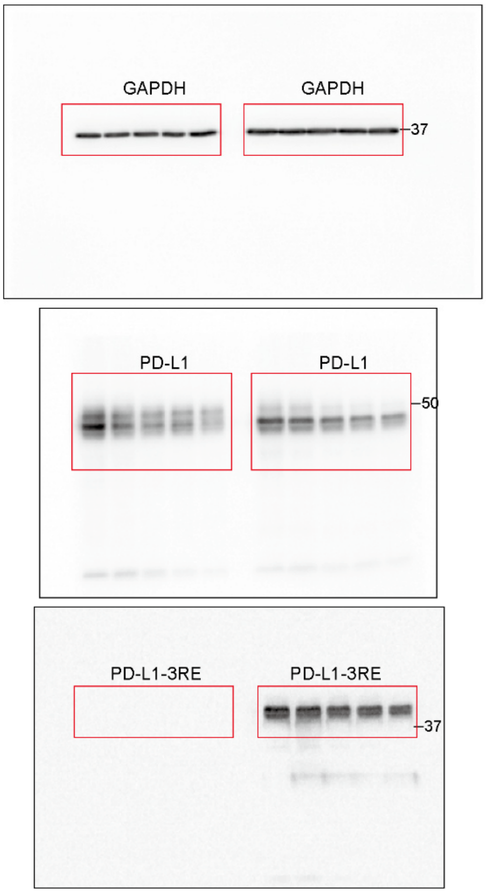


Fig.6f

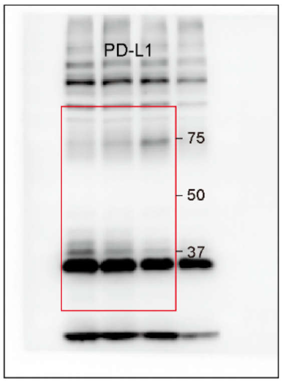

Fig.7a

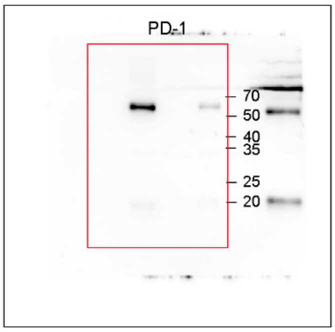


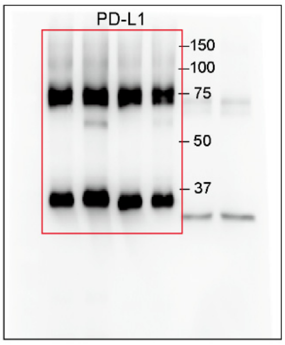


Fig.7b

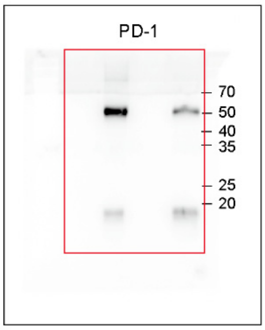


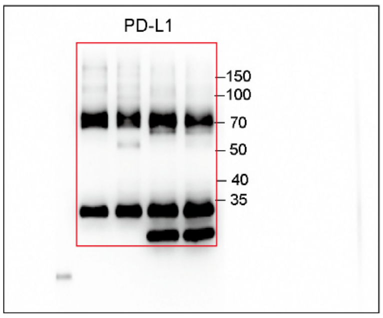


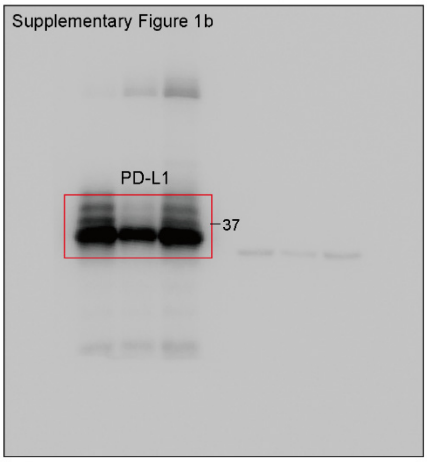


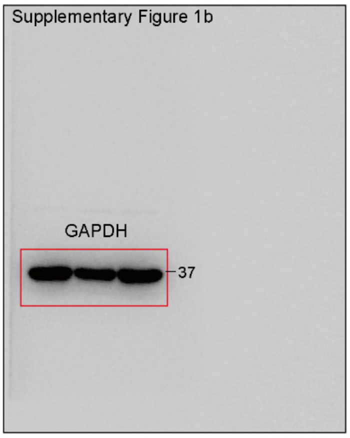


Supplementary Figure 1c

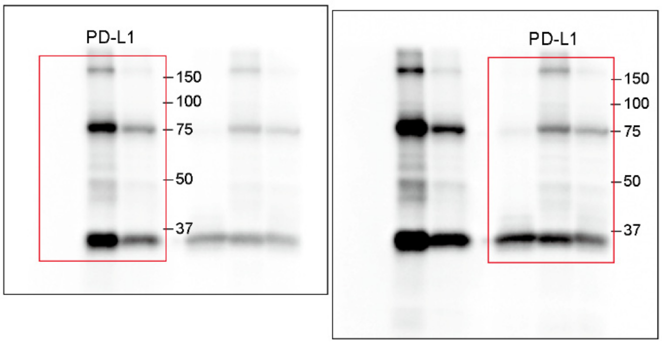


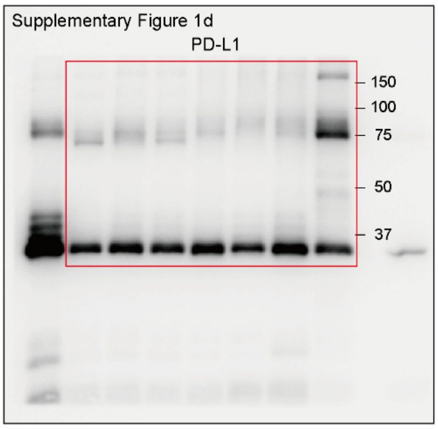

Supplementary Figure 2a

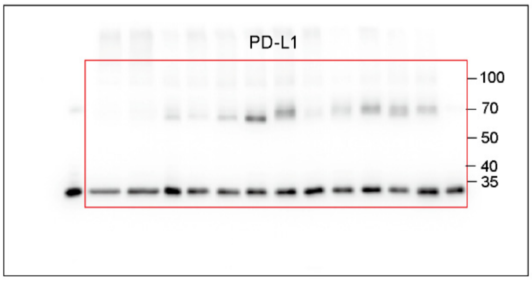


Supplementary Figure 2b

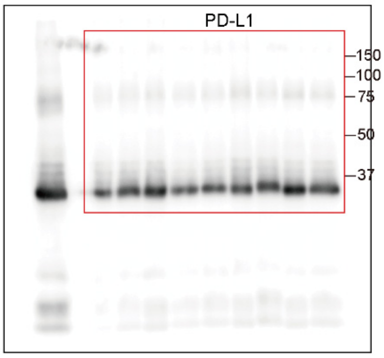


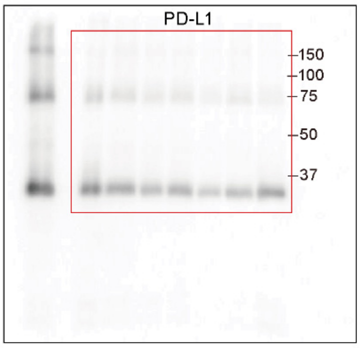


Supplementary Figure 2c

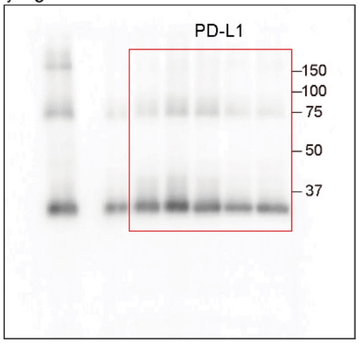


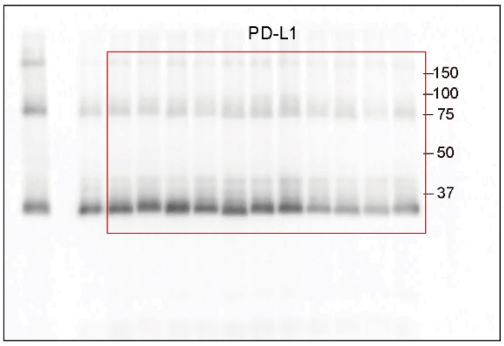


Supplementary Figure 3a

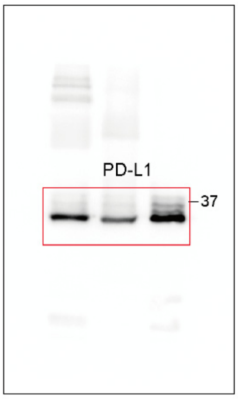


Supplementary Figure 3c

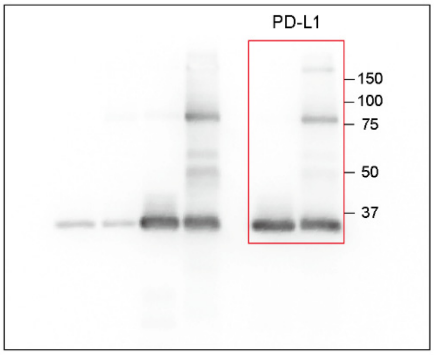


Supplementary Figure 3d

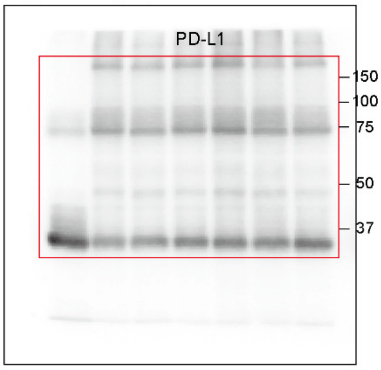

Supplementary Figure 3e

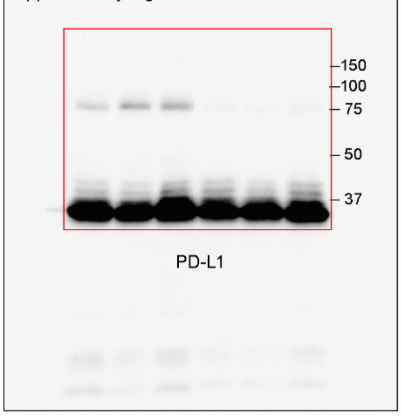


Supplementary Figure 3f

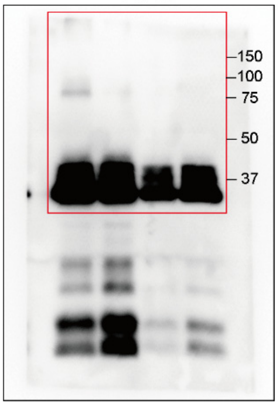


Supplementary Figure 4a

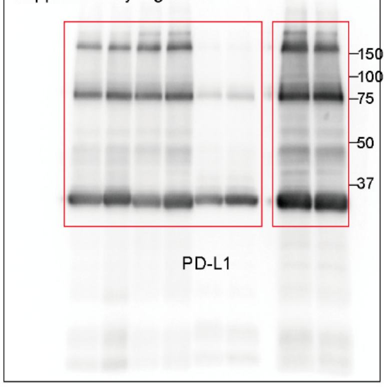


Supplementary Figure 4a

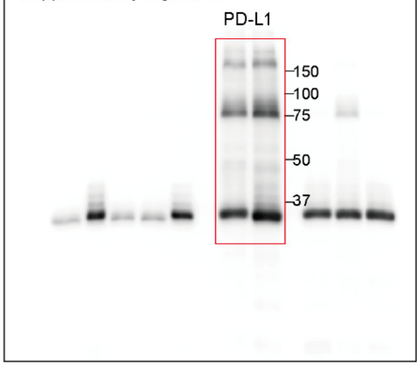


Supplementary Figure 4b

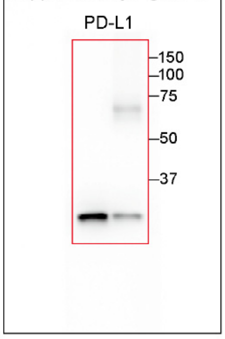


Supplementary Figure 4c

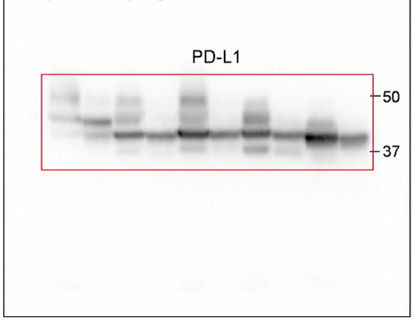


Supplementary Figure 4c

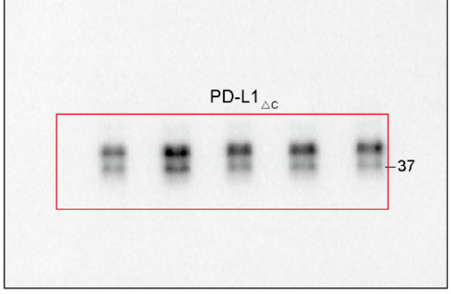


Supplementary Figure 5a

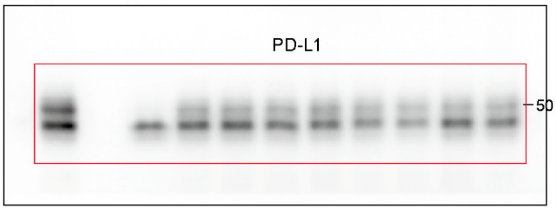


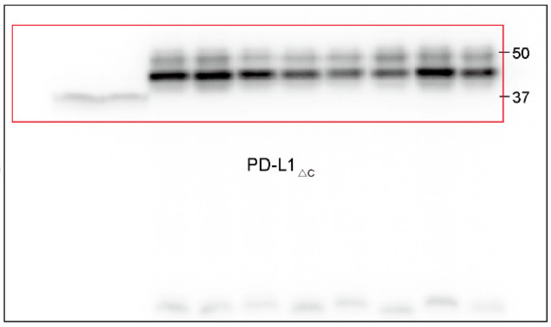

Supplementary Figure 5b

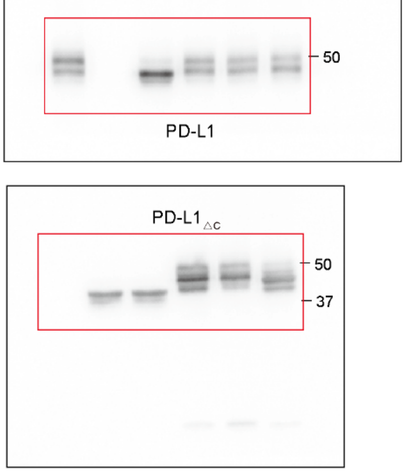


Supplementary Figure 5c

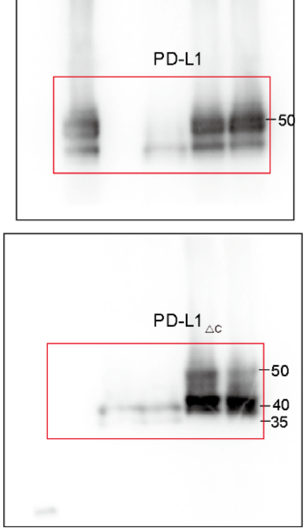


Supplementary Figure 5d

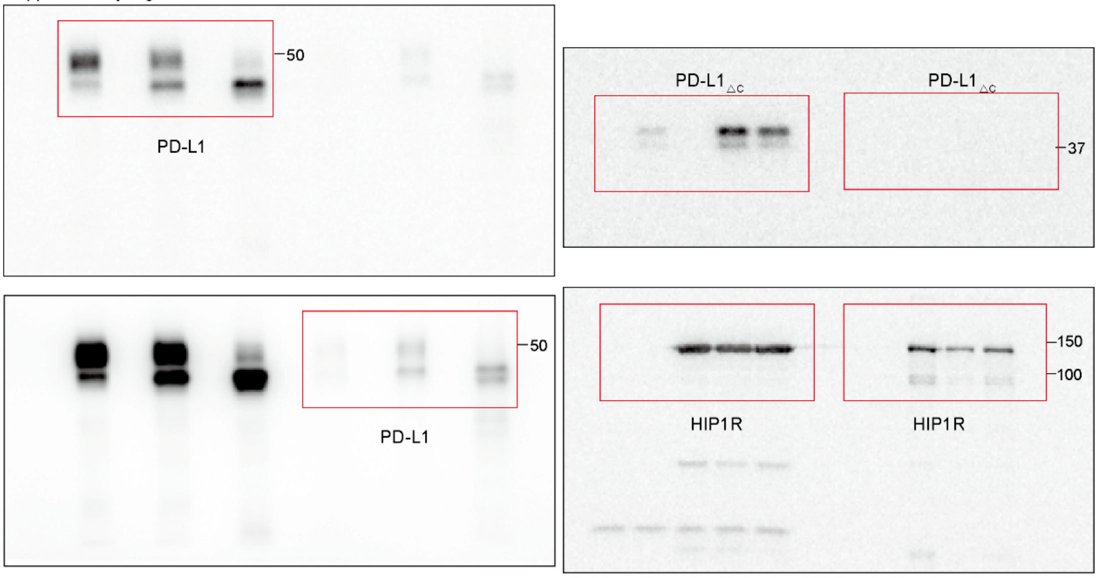


Supplementary Figure 7a

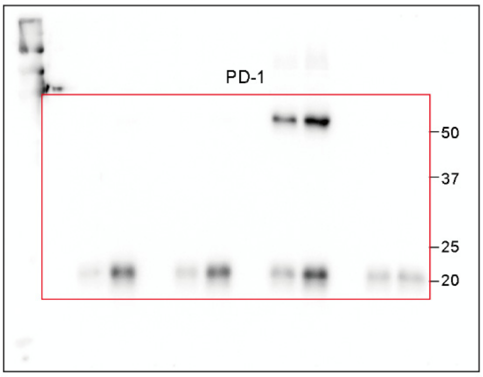


Supplementary Figure 7b

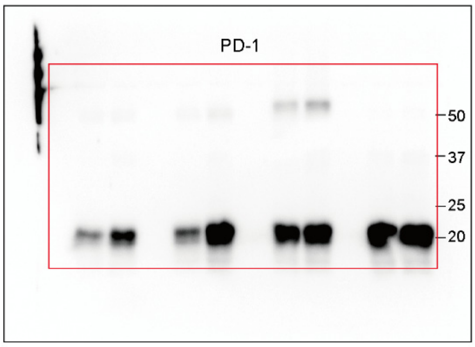

Supplementary Figure 7c

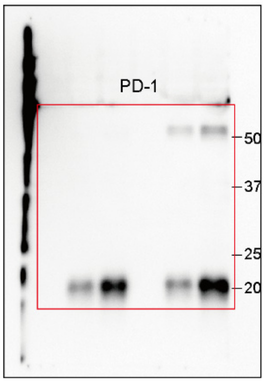


Supplementary Figure 7d

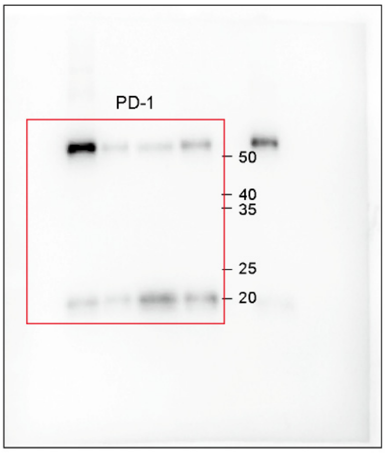


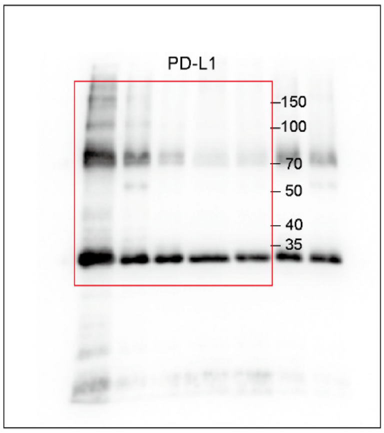

Supplement: Supplementary file 1 — Supplementary Information [file 42003_2022_3845_MOESM1_ESM.pdf]
