# Supplementary material for: Homodimerized cytoplasmic domain of PD-L1 regulates its complex glycosylation in living cells
Source: Commun Biol. 2022 Aug 30;5:887. doi: 10.1038/s42003-022-03845-4 (PMC9427764; doi:10.1038/s42003-022-03845-4)
Supplement: Supplementary file 2 — Description of Additional Supplementary Files [file 42003_2022_3845_MOESM2_ESM.pdf]

Description of Additional Supplementary Files

File name: Supplementary Data 1
Description: Source data for Figure 6e and Figure 7d.
