# Supplementary material for: Homodimerized cytoplasmic domain of PD-L1 regulates its complex glycosylation in living cells
Source: Commun Biol. 2022 Aug 30;5:887. doi: 10.1038/s42003-022-03845-4 (PMC9427764; doi:10.1038/s42003-022-03845-4)
Supplement: Supplementary file 4 — Reporting Summary [file 42003_2022_3845_MOESM4_ESM.pdf]

nature portfolio

Corresponding author(s): Haiyan Ren

Last updated by author(s): Aug 1, 2022

Reporting Summary

Nature Portfolio wishes to improve the reproducibility of the work that we publish. This form provides structure for consistency and transparency in reporting. For further information on Nature Portfolio policies, see our Editorial Policies and the Editorial Policy Checklist.

Statistics

For all statistical analyses, confirm that the following items are present in the figure legend, table legend, main text, or Methods section.

n/a	Confirmed	
☐	☒	The exact sample size (*n*) for each experimental group/condition, given as a discrete number and unit of measurement
☐	☒	A statement on whether measurements were taken from distinct samples or whether the same sample was measured repeatedly
☐	☒	The statistical test(s) used AND whether they are one- or two-sided<br>*Only common tests should be described solely by name; describe more complex techniques in the Methods section.*
☒	☐	A description of all covariates tested
☒	☐	A description of any assumptions or corrections, such as tests of normality and adjustment for multiple comparisons
☐	☒	A full description of the statistical parameters including central tendency (e.g. means) or other basic estimates (e.g. regression coefficient) AND variation (e.g. standard deviation) or associated estimates of uncertainty (e.g. confidence intervals)
☐	☒	For null hypothesis testing, the test statistic (e.g. *F*, *t*, *r*) with confidence intervals, effect sizes, degrees of freedom and *P* value noted<br>*Give P values as exact values whenever suitable.*
☒	☐	For Bayesian analysis, information on the choice of priors and Markov chain Monte Carlo settings
☒	☐	For hierarchical and complex designs, identification of the appropriate level for tests and full reporting of outcomes
☒	☐	Estimates of effect sizes (e.g. Cohen's *d*, Pearson's *r*), indicating how they were calculated

Our web collection on statistics for biologists contains articles on many of the points above.

Software and code

Policy information about availability of computer code

Data collection	No custom code used.<br>ZAISS LSM 880 was controlled by ZEN SP1 (v. 2.3).
Data analysis	No custom code used.<br>Basic statistical analysis was performed using GraphPad Prism 5 (v. 5.01). Figures and plots were made using Adobe illustrator cc2019.<br>Western blot band quantification were done using ImageJ (v. 1.37c). Protein structure plots were made using PyMOL (v. 1.5.x).<br>Immunofluorescence image processing was done using ZEN 2012 (v. 1.1.0001).

For manuscripts utilizing custom algorithms or software that are central to the research but not yet described in published literature, software must be made available to editors and reviewers. We strongly encourage code deposition in a community repository (e.g. GitHub). See the Nature Portfolio guidelines for submitting code & software for further information.

Data

Policy information about availability of data

All manuscripts must include a data availability statement. This statement should provide the following information, where applicable:

- Accession codes, unique identifiers, or web links for publicly available datasets
- A description of any restrictions on data availability
- For clinical datasets or third party data, please ensure that the statement adheres to our policy

Plasmids used in this study and other materials are available upon reasonable request. All data supporting the findings of this study are available within the paper and the supplementary information or from the corresponding author upon reasonable request.

Field-specific reporting

Please select the one below that is the best fit for your research. If you are not sure, read the appropriate sections before making your selection.

☒ Life sciences ☐ Behavioural & social sciences ☐ Ecological, evolutionary & environmental sciences

For a reference copy of the document with all sections, see nature.com/documents/nr-reporting-summary-flat.pdf

Life sciences study design

All studies must disclose on these points even when the disclosure is negative.

Sample size	Experiments were done with independent biological samples (usually n≥3) and highly comparable results were obtained. This sample size is generally accepted as sufficient when using independent biological samples.
Data exclusions	No data was excluded.
Replication	All attempts to replicate were successful.
Randomization	Not applicable to this study.
Blinding	Not applicable to this study.

Reporting for specific materials, systems and methods

We require information from authors about some types of materials, experimental systems and methods used in many studies. Here, indicate whether each material, system or method listed is relevant to your study. If you are not sure if a list item applies to your research, read the appropriate section before selecting a response.

Materials & experimental systems

n/a	Involved in the study	
☐	☒	Antibodies
☐	☒	Eukaryotic cell lines
☒	☐	Palaeontology and archaeology
☒	☐	Animals and other organisms
☒	☐	Human research participants
☒	☐	Clinical data
☒	☐	Dual use research of concern

Methods

n/a	Involved in the study	
☒	☐	ChIP-seq
☒	☐	Flow cytometry
☒	☐	MRI-based neuroimaging

Antibodies

Antibodies used	rabbit anti-PD-L1 (13684, CST; WB 1:2000), rabbit anti-HA (ab9110, Abcam; IF 1:400), mouse anti-HA (M20003, Abmart; WB 1:2000), mouse anti-flag (M20008, Abmart; IF 1:400, WB 1:2000), rabbit anti-Strep ? (HA500061, HuaBio; WB 1:2000), mouse anti-GAPDH (AC002, ABclonal; WB 1:4000), mouse anti-PDI (MA3-019, Thermofisher; IF 1:100), mouse anti-GM130 (610822, BD; IF 1:100), HRP-conjugated goat anti-Rabbit IgG(H+L) (SA00001-2, Proteintech; WB 1:2000), HRP-conjugated goat anti-Mouse IgG (H+L) (31430, Invitrogen; WB 1:2000), goat anti-Rabbit IgG(H+L) cross-adsorbed Alexa Fluor 488 (A-11008, Thermofisher; IF 1:400), goat anti-Mouse IgG(H+L) highly cross-adsorbed Alexa Fluor Plus 647 (A32728, Thermofisher; IF 1:400)
Validation	All antibodies are validated for the application used in this study according to the manufacturers' websites.

Eukaryotic cell lines

Policy information about cell lines

Cell line source(s)	HEK293T cells were purchased from National Collection of Authenticated Cell Cultures, RKO cells were purchased from iCell Bioscience Inc.
Authentication	Cells obtained from ATCC and iCell Bioscience Inc had been authenticated by the supplier.
Mycoplasma contamination	All cell lines tested negative for mycoplasma contamination based on the PCR assays.
Commonly misidentified lines (See ICLAC register)	No commonly misidentified cell lines were used.
